# Direct Observation of Triplet–Triplet Energy Transfer in DNA between Energy Donor and Acceptor C‑Nucleotides

**DOI:** 10.1021/jacsau.5c00364

**Published:** 2025-05-27

**Authors:** Sebastian Häcker, Till J. B. Zähringer, Hans-Achim Wagenknecht, Christoph Kerzig

**Affiliations:** † Institute of Organic Chemistry, Karlsruhe Institute of Technology (KIT), Karlsruhe 76131, Germany; ‡ Department of Chemistry, Johannes Gutenberg University Mainz, Duesbergweg 10-14, Mainz 55128, Germany

**Keywords:** DNA, energy transfer, oligonucleotides, photochemistry, time-resolved spectroscopy

## Abstract

Investigating the migration of excited-state energy in DNA is crucial for a deep understanding of protection mechanisms and light-induced DNA damage. While numerous reports focused on single electron transfer and Förster-type energy transfer in DNA, studies on the Dexter-type triplet–triplet energy transfer are scarce, in particular, those with direct detection of photoexcited triplet states. Herein, we present direct measurements of the distance-dependent triplet–triplet energy transfer rates through DNA by using transient absorption spectroscopy. This was achieved through the synthetic incorporation of thioxanthone as an energy donor and naphthalene as an energy acceptor into a DNA double strand at defined positions. The energy transfer rates strongly depend on the number of A-T base pairs (up to four) separating the energy donor from the energy acceptor. We observed a fast energy transfer rate with a time constant of 17 ns for the DNA sample in which the donor and acceptor are directly adjacent in the DNA. By analyzing two additional donor–acceptor distances, a steep exponential distance dependence with an attenuation factor of 1.15 Å^–1^ could be obtained. Our results demonstrate that DNA acts as a poor conductor of triplet energy when energy donors with triplet energies below 2.7 eV are used, complementing more indirect studies on sensitized DNA damage.

## Graphical abstract

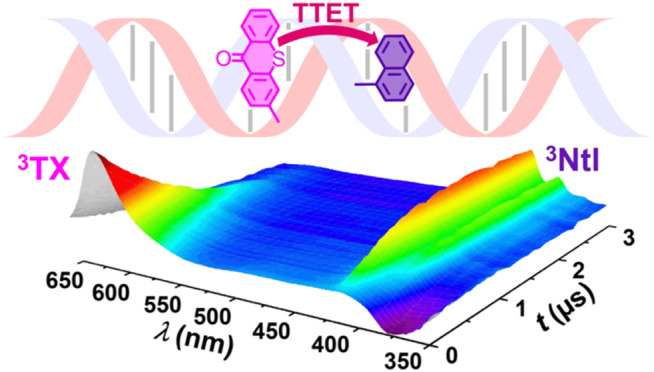

## Introduction

Solar ultraviolet (UV) light can be a threat to DNA, leading to light-induced DNA damage which, in the worst case, can lead to skin cancer.^1–3^ Nonetheless, in most cases, the excited states in DNA decay extremely fast (<1 ps) into charge-separated states. These return to their ground state through charge recombination, a property of DNA that protects it from light-induced damage.^4–6^ However, excited singlet states can also lead to photochemical reactions. For example, cyclobutane pyrimidine dimers (CPDs) are the most common DNA damage and are formed by singlet photochemistry on ultrafast time scale and in a nearly barrierless reaction at the site of excitation.^7–9^ In addition to the well-studied singlet photochemistry in DNA, it has been shown that excited triplet states also play an important role in the formation of DNA damage such as one-electron oxidation, cross-linking or CPDs.^10–15^ Singlet energy transfer typically occurs via dipole–dipole interactions, known as Förster Resonance Energy Transfer (FRET),^16^ from an excited donor to an acceptor. In contrast, triplet–triplet energy transfer (TTET) proceeds predominantly through the exchange mechanism (Dexter-type),^17^ which shares mechanistic similarities with electron transfer.^18^ Dexter energy transfer requires orbital overlap and, therefore, close proximity between the donor and acceptor. The range of triplet energy transfer can be significantly extended by introducing bridging units that enable through-bond energy transfer,^19^ a phenomenon extensively studied in molecular systems.^20,21^ In certain cases, triplet-to-singlet energy transfer via dipole–dipole interactions has also been observed,^22^ or both mechanisms may operate in tandem.^23^ Depending on the thermodynamic barriers introduced by the bridging units, energy transfer may occur sequentially through intermediate states via a hopping mechanism, provided these states are energetically accessible, or through tunneling. The mechanism by which triplet energy transfer in DNA leads to DNA damage is not yet fully understood, primarily because it is challenging to excite and localize triplet states in DNA at defined positions. Yet understanding the different photochemical mechanisms leading to DNA damage is crucial for elucidating the different pathways to skin cancer.

In recent studies, triplet states of DNA bases have been made accessible by photosensitizers, which were selectively incorporated into the DNA as C-nucleosides. By indirect detection methods, we observed distance-dependent energy transfer in the DNA leading to CPD damage.^24–26^ Localized triplet states of DNA bases have been spectroscopically observed,^27–29^ and emission-based measurements involving DNA-intercalated metal complexes as energy donors suggest the occurrence of triplet–triplet energy transfer.^30,31^ However, the observed shallow distance dependence of energy migration deviates from the predictions of the exchange mechanism,^30,31^ instead aligning more closely with long-range Förster transfer^32,33^ or a combination of both mechanisms.^30^ This highlights the limitations of relying on indirect, emission-based techniques, which can introduce ambiguity regarding the underlying mechanism, particularly in complex systems like DNA.^34^ Another study led by Brun and Harriman used intercalated acridine orange as energy donor tracking energy transfer to a palladium complex by transient absorption methods.^35^ However, a definitive conclusion is challenging, as dipole–dipole interactions occurred simultaneously, and the lack of covalent bonding between the energy donor and acceptor further complicated the interpretation of the results. To the best of our knowledge, we provide the first direct evidence of an exchange-type triplet energy transfer through DNA, demonstrated using transient absorption spectroscopy. Our study thus complements prior experimental and theoretical studies on triplet states and photosensitization in DNA.^36–43^

## Results and Discussion

### Design of the Study

We selected thioxanthone (TX) as the triplet energy donor and naphthalene (Ntl) as the triplet energy acceptor. This extensively studied donor–acceptor pair has well-established triplet–triplet absorption spectra (see Scheme 1) and energy transfer kinetics, making it a textbook example for triplet–triplet energy transfer.^44^ Moreover, the long triplet state lifetimes of both the donor and acceptor chromophore (>10 μs),^45^ which is typical for organic chromophores, facilitate the detection of slower quenching processes anticipated at greater donor–acceptor distances. The triplet state energy of thioxanthone is reported at 2.75 eV^46^ giving the required thermodynamic driving force for the energy transfer to naphthalene (2.63 eV).^45^ Notably, this energy should be insufficient to directly sensitize the DNA bases by triplet–triplet energy transfer,^40,47^ in alignment with our prior study monitoring CPD damage with a thioxanthone C-nucleoside as the sensitizer.^26^ These characteristics make the TTET donor–acceptor pair an ideal candidate for studying triplet energy migration in DNA in a direct fashion. In this study, the triplet energy donor thioxanthone and the triplet energy acceptor naphthalene were integrated into complementary DNA strands, both as C-nucleosides at specific distances using phosphoramidite chemistry. We investigate three DNA double strands abbreviated as **TX-Ntl-*n***, along with three reference DNA samples labeled **TX-*n*** (where *n* = 0, 1, 2), corresponding to the number of two alternating A-T base pairs separating the TX and Ntl C-nucleosides (see Scheme 1). The reference samples were synthesized under identical conditions, however, at the position of the Ntl C-nucleoside, the initial base (thymine) is reinstalled. This allows us to directly assess the presence and absence of the energy acceptor on the photochemistry of the TX C-nucleoside.

**Scheme 1:**
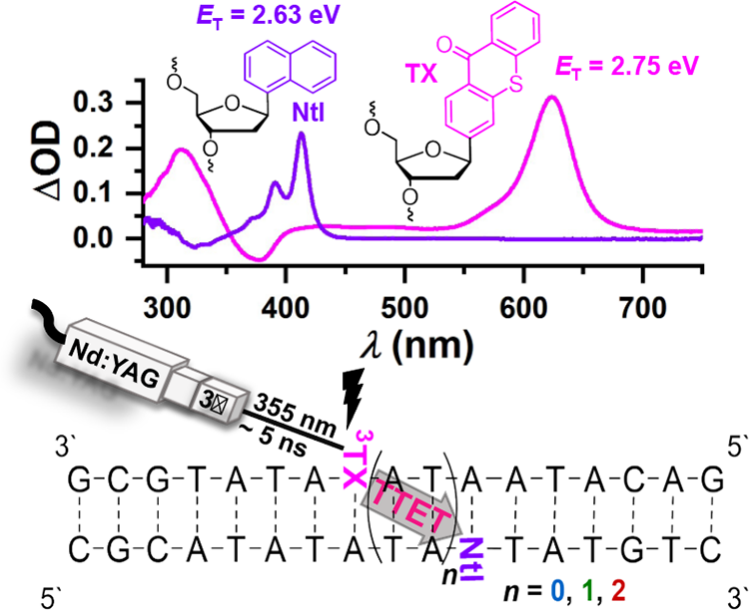
Top: Structure of Energy Donor (TX) and Acceptor (Ntl) Containing C-Nucleosides and Color-Coded Reference Triplet–Triplet Absorption Spectra Obtained for the Isolated Chromophores in Acetonitrile upon 355 nm Laser Excitation^a^

a Bottom: Schematic representation of the DNA double strands **TX-Ntl-*n*** used to analyze the distance dependence of the TTET kinetics in DNA double strands.

### Synthesis

The synthesis of the corresponding thioxanthone C-nucleoside as a DNA building block for our study was reported previously by one of our groups. The naphthalene C-nucleoside **4** was obtained by a similar synthetic approach.^26^ Briefly, the first and key step is a Heck reaction. The TBDMS-protected glycal **1** for this reaction was obtained from TBDMS-protected thymidine by elimination of thymine, while 1-bromonaphthalene was available from a commercial supplier.^48^ In this case the catalyst Pd_2_(dba)_3_ with the ligand Q-Phos proved to be most effective with a combined yield of 79% of singly and doubly TBDMS-protected product **2**. The TBDMS group in the 2’-position shields the down face of the glycal **1**. As a result, the Heck reaction occurs stereoselectively to the desired and natural-like β-anomer **2**. The further reactions are deprotection of the TBDMS groups of **2**, stereoselective reduction of the carbonyl group at the 3′-position of **3** and conversion of **4** in two steps to the desired phosphoramidite **6** were carried out following our previously established procedures (Scheme 2).^26^

**Scheme 2:**
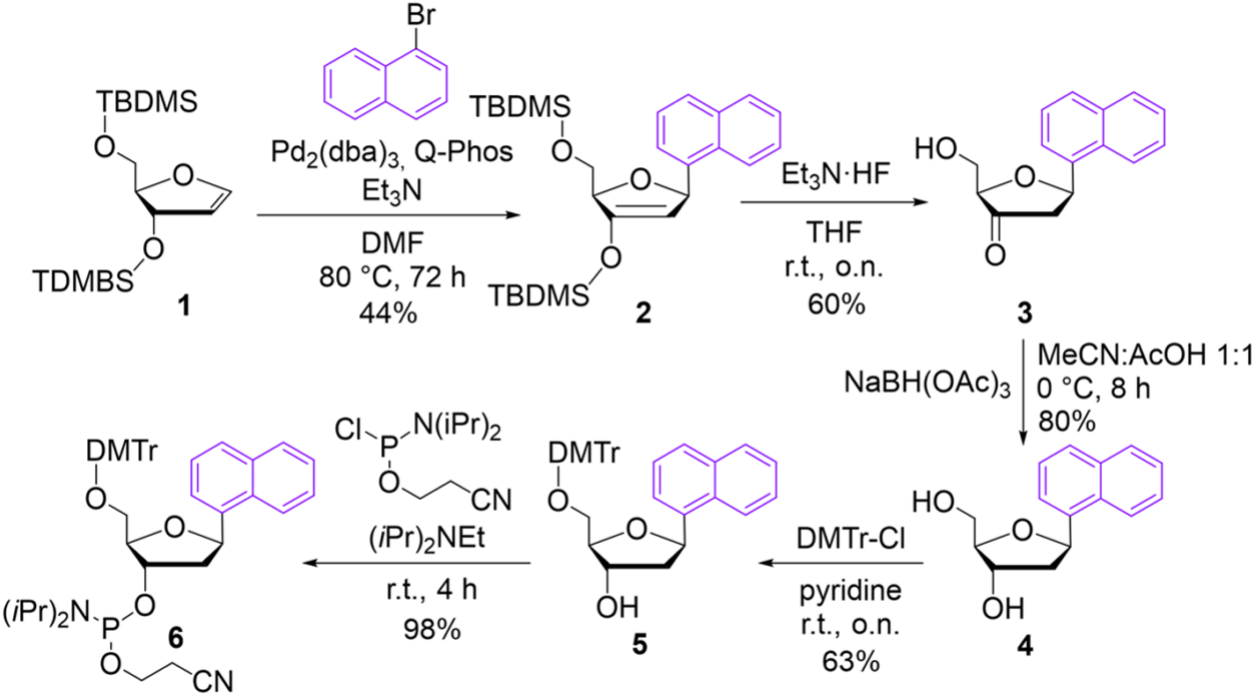
Synthesis of Naphthalene C-Nucleoside **4** and Phosphoramidite **6** as DNA Building Block

The DNA double strands **TX-Ntl-0**, **TX-Ntl-1** and **TX-Ntl-2** were prepared using the TX- and Ntl-containing phosphoramidites as DNA building blocks. Both phosphoramidites were characterized by ^1^H and ^31^P NMR spectroscopy as well as HR ESI-MS (Figures S27–S29). The resulting DNA single strands were synthesized via automated oligonucleotide solid-phase synthesis and purified by RP-HPLC through fractional collection. Fractions containing the desired DNA strand, determined by MALDI-TOF-MS, were combined, and the purity was confirmed again using analytical RP-HPLC (Figures S32–S34). The complementary DNA single strands modified either with TX or Ntl were annealed to double strands by heating to 90 °C for 10 min and slow cooling to room temperature. As control DNA samples, double strands were prepared without the triplet energy acceptor naphthalene (Figure 1, top). Furthermore, a reference DNA sample containing only Ntl (**Ntl-Control**) was prepared (i.e., without TX; see Figure 1 for its structure). The unmodified DNA single strands for the latter double strands were purchased.

**Figure 1:**
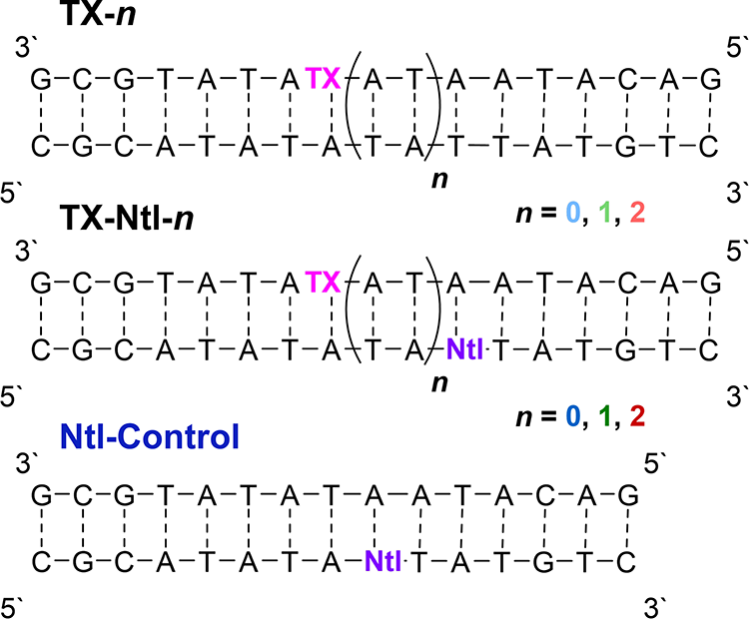
Top: Sequences of DNA double strands **TX-*n*** modified with TX and **TX-Ntl-*n*** modified with both TX and Ntl. Bottom: color-coded UV–vis absorption spectra of 90 μM DNA double strands **TX-*n***, **TX-Ntl-*n*** and **Ntl-Control** (dark blue) in aqueous buffer solution (250 mM NaCl, 10 mM Na–P_i_ buffer, pH 7.0) and of 80 μM thioxanthone (pink) or 200 μM naphthalene (purple) in MeCN.

### Optical Spectroscopy

The UV–vis absorption spectra of all DNA double strands show ∼ 20 nm red shifts of the long-wavelength absorption band of thioxanthone to 397 nm compared to the absorption spectrum of thioxanthone in acetonitrile with a maximum at 378 nm (Figure 1, bottom). The observed red shift can be explained by the more polar DNA environment, assuming that the TX chromophore is intercalated in the DNA base stack. This leads to a stabilization of the S_1_ (π-π*) state.^49^ As a result, a relatively high concentration (90 μM, based on double-strand DNA) of the samples was required to ensure efficient excitation at our laser wavelength 355 nm and sensitive TA detection accordingly. As is evident from the reference spectra of naphthalene in MeCN and the DNA double strand **Ntl**-**Control** (see Figure 1), both the naphthalene chromophore and the conventional DNA bases do not absorb at 355 nm, ensuring selective laser excitation of TX (see Section S4.2 for further control experiments).

Transient absorption spectroscopy employing a pulsed 355 nm laser (∼20 mJ, ∼5 ns pulse duration) was used to excite the DNA double strands in deaerated aqueous solution containing 250 mM NaCl and 10 mM Na–P_i_ buffer (buffer pH 7: 6.1 mM sodium phosphate monobasic and 3.9 mM sodium phosphate dibasic). The first sample we investigated was **TX-Ntl-0**, in which TX and Ntl are adjacent without any base pairs in between, together with the reference DNA double strand **TX-0**. We first analyzed the TA spectrum of **TX-0**. Immediately following excitation (100 ns), two absorption bands appeared at 320 and 612 nm, along with a ground-state bleach (GSB) observed at ∼ 400 nm (Figure 2A).

**Figure 2:**
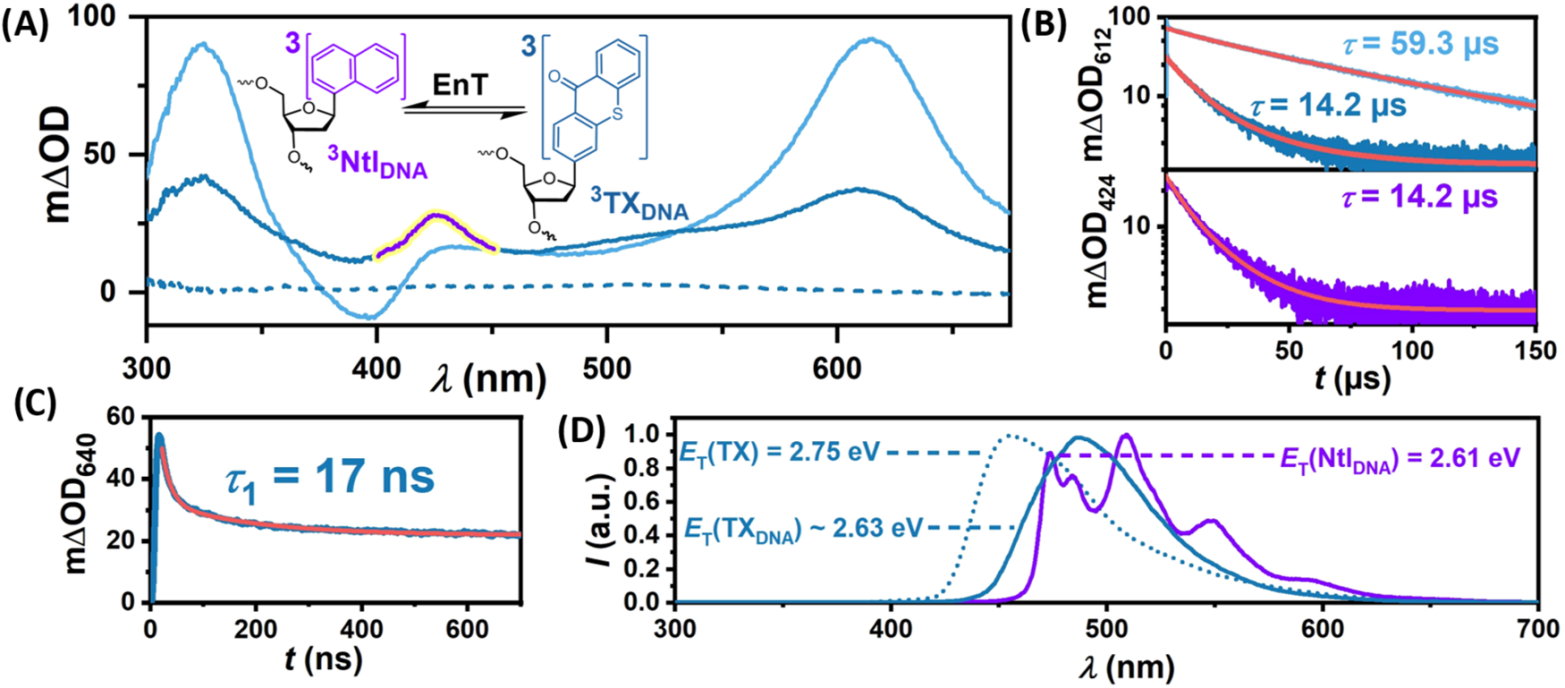
Mechanistic studies of **TX-Ntl-0** (dark blue) and **TX-0** (light blue) with 355 nm laser pulses. (A) Transient absorption spectra recorded 100 ns after excitation. The triplet–triplet absorption band of Ntl_DNA_ is highlighted in purple. (B) and (C) Time-resolved measurements at different detection wavelengths and time scales. (D) Time-gated 77 K emission of TX in MeCN (dotted line) and **TX-Ntl-0** in aqueous buffer (250 mM NaCl, 10 mM Na–P_i_ buffer, pH 7.0) integrated 10 ms – 100 ms (blue) and 4.0 to 4.3 s (purple) after laser excitation.

These bands are associated with a TX-localized triplet state of the C-nucleotide of TX (^3^TX_DNA_),^46^ which is rapidly generated via intersystem crossing from the singlet-excited state.^50^ Compared to the triplet absorption spectrum of TX in MeCN (Figure S1A), the bands exhibit slight shifts, a phenomenon previously noted for TX in protic solvents and attributed to substitution at position 2.^51^ We obtain a triplet state lifetime of 59.3 μs for **TX-0** (Figure 2B, top panel), within the lifetime range of unquenched ^3^TX and its derivatives in aprotic and protic solvents.^52–54^ We conclude that the triplet-excited state of the C-nucleotide of TX is not quenched by the DNA bases in line with prior investigations, where CPD as a result of triplet–triplet energy transfer was only observed for triplet energy donors with higher triplet energies (>2.80 eV).^26^ Exciting the DNA **TX-Ntl-0** yielded an additional absorption band centered at 424 nm alongside the triplet absorption spectrum of TX directly after excitation (a 100 ns delay time was used to ensure the absence of stray light and fluorescence). This absorption band could be attributed to the Ntl-localized triplet state of the C-nucleotide (^3^Ntl_DNA_). Compared to the triplet absorption spectrum of Ntl in MeCN (Figure S1A), the fine structure is lost due to broadening and the spectrum is shifted to longer wavelengths. However, these observations can be traced back to the solvent influence on the Ntl triplet^55^ and to the conversion of Ntl to the 2′-deoxyribofuranoside at the α-position.^56^ In line with our observations, Kiefhaber et al. reported a similar transient absorption spectrum for ^3^Ntl, with a peak at 420 nm, when they investigated the TTET dynamics between TX and Ntl connected by a peptide bridge.^57^ We found that both transient species concurrently decay with a lifetime of 14.2 μs (Figure 2B). Assigning the species with the ∼420 nm absorption band to ^3^Ntl_DNA_ raises the question of why both ^3^Ntl_DNA_ and ^3^TX_DNA_ appear to form after excitation (100 ns delay) and decay with essentially identical lifetimes. This is particularly puzzling given that (i) we have ruled out the direct excitation of Ntl_DNA_ and (ii) the proposed energy transfer mechanism suggests that ^3^Ntl_DNA_ should be generated during the quenching of ^3^TX_DNA_. Indeed, the C-nucleotides of TX and Ntl in the **TX-Ntl-0** DNA are in close proximity (3.4 Å based on regular stacking distance),^58^ which is within the typical distance required for efficient Dexter-type energy transfer (<1 nm). Consequently, we expect the energy transfer to occur nearly instantaneously (<100 ns). However, detection in that range is challenging due to the strong fluorescence signal from TX (see also Section S4.4), which causes detector saturation. For this reason, a detection wavelength of 640 nm was chosen for ^3^TX_DNA_ (Figure 2C), where the TX singlet state does not emit anymore. Time-resolved measurements show indeed a more complex kinetic scenario, and a biexponential fit resulted in a lifetime of 17 ns for the faster component, superimposed by the longer lifetime (compare Figure 2B). The seemingly incomplete quenching of ^3^TX_DNA_ prompted us to conduct time-gated 77 K emission spectroscopy. Interestingly, we observed two emission signals at different time intervals, which can be attributed to the phosphorescence of ^3^TX_DNA_ followed by that of ^3^Ntl_DNA_ (Figure 2D). Compared to the phosphorescence of TX in MeCN, the emission from the thioxanthone C-nucleoside is significantly red-shifted (∼40 nm), indicating a decrease in triplet state energy. Similar environment effects on triplet-related properties in DNA were predicted and observed before.^26,59,60^

We propose that the lower triplet state energy of TX_DNA_ reduces the energy gap to ^3^Ntl_DNA_, which enables back energy transfer (bTTET) at room temperature reaching an equilibrated state.^61–63^ At 77 K bTTET becomes negligibly slow as is evident from time-gated emission spectra shown in Figure S11. Based on all these observations, we estimate the triplet state energy of Ntl_DNA_ to be 2.61 eV. Determining the triplet energy of TX_DNA_ is more challenging due to its broad phosphorescence spectrum. However, using the relative distribution of triplet states between Ntl_DNA_ and TX_DNA_ at 77 K and in the equilibrated state at room temperature, we estimate its energy to be approximately 2.63 eV (see Section S4.7 of the SI for details). These energy estimations support the assumption that TX has a lower triplet state energy than the DNA bases, consistent with the absence of characteristic transient absorption bands from the nucleotides.^40^ Emission quenching of singlet-excited TX_DNA_ seems to be a minor process and occurs on a shorter time scale than the observed initial ^3^TX_DNA_ decay and ^3^Ntl_DNA_ formation (Section S4.4), ruling out significant contributions of Förster-like energy transfer processes. To summarize the results obtained for DNA **TX-Ntl-0**, after the formation of ^3^TX_DNA_ we unequivocally observe rapid triplet–triplet energy transfer to Ntl_DNA_ with *k*_TTET_ = (58.8 ± 11.7) × 10^6^ s^–1^ (see SI for calculation of *k*_TTET_), reaching an equilibrated state^64^ of ^3^TX_DNA_ and ^3^Ntl_DNA_ owing to the almost isoenergetic triplet states. These triplet states decay on a much longer time scale (*k*_TTET_, *k*_bTTET_ ≫ *k*_TX_, *k*_Ntl_). Interestingly such an energy transfer equilibrium has also been described by Kiefhaber et al. for the same donor–acceptor pair in a peptide environment.^57^

Next, we examined the DNA double strand **TX-Ntl-1**, with two A-T base pairs separating the TX and Ntl C-nucleotides, in conjunction with the reference DNA **TX-1**. The TA spectra recorded 100 ns after 355 nm laser excitation are almost identical for these DNA samples (Figure 3A).

**Figure 3:**
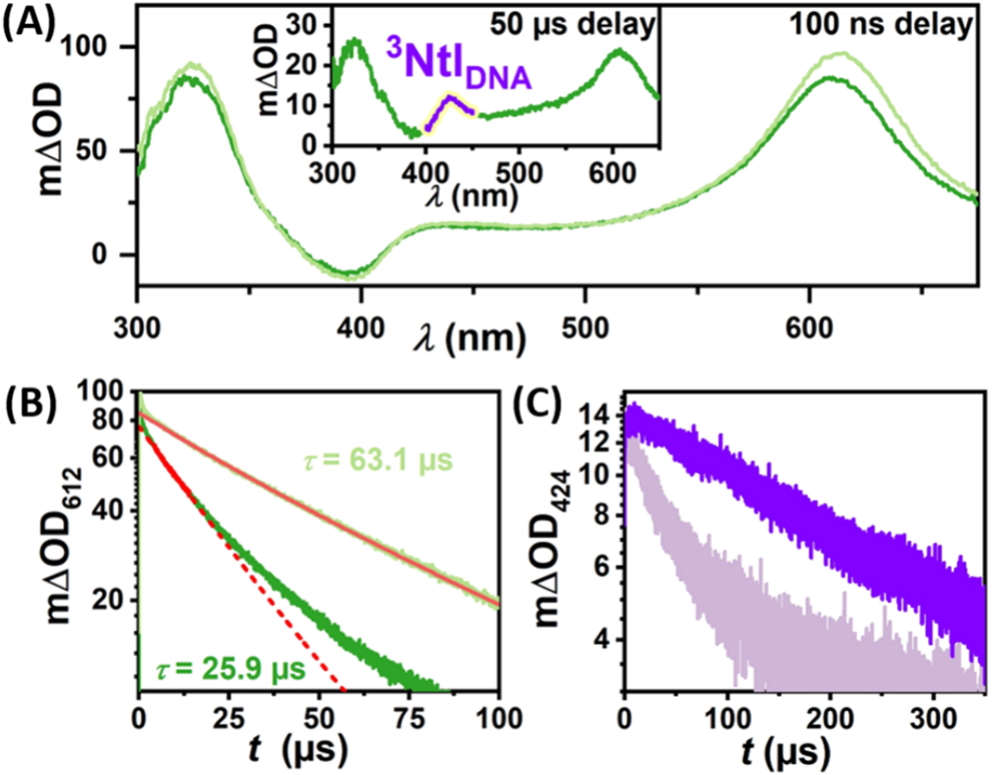
Mechanistic studies of **TX-Ntl-1** (dark green) and **TX-1** (light green) with 355 nm laser pulses. (A) TA spectra recorded 100 ns after excitation. Inset: TA spectrum of **TX-Ntl-1** recorded 50 μs after excitation. The triplet–triplet absorption band of Ntl_DNA_ is highlighted in purple. (B) Kinetic TA measurements at 612 nm. (C) Kinetic TA measurements of **TX-Ntl-1** (purple) and **TX-1** (light purple) at 424 nm.

Yet, time-resolved measurements of ^3^TX_DNA_ at 612 nm, where it can be monitored in isolation, reveal a faster deactivation in the presence of Ntl_DNA_ (**TX-Ntl-1**) in comparison to its absence (**TX-1**). This observation suggests ^3^TX_DNA_ quenching by Ntl_DNA_ (Figure 3B). Indeed, we observe the formation of the ^3^Ntl_DNA_ band at longer delay times (inset of Figures 3A, and S9). Comparative kinetic measurements at 424 nm show the slow formation of ^3^Ntl_DNA_ in **TX-Ntl-1** (Figure 3C). This signal rise cannot be observed for **TX-1**, which again shows a lifetime being typical for unquenched ^3^TX. For this rather slow quenching process, we ruled out the possibility of inter-DNA energy transfer (from TX of one DNA double strand to Ntl in another DNA double strand, compare results with **TX-Ntl-2** presented below) and attribute this to intra-DNA triplet energy migration from ^3^TX_DNA_ to Ntl_DNA_ (in the same DNA double strand). Moreover, ^3^TX_DNA_ was only moderately quenched by dissolved oxygen in the air-saturated solution of **TX-0**, underscoring the protective geometry of the DNA backbone. To determine the energy transfer constant, the decay trace of ^3^TX_DNA_ at 612 nm was fitted monoexponentially until the energy transfer efficiency η_EnT_ reaches ∼ 30% and back energy transfer has to play a minor role, making the reasonable assumption that TTET is faster than bTTET. Using the unquenched (τ = 63.1 μs) and quenched lifetime of ^3^TX_DNA_ (τ = 25.9 μs) we calculate an energy transfer rate constant of *k*_TTET_ = (22.7 ± 4.0) × 10^3^ s^–1^. In contrast to **TX-Ntl-0** the (back) energy transfer constant in **TX-Ntl-1** is several orders of magnitude slower due to the larger separation of the C-nucleotides in the DNA (10.2 Å), and on the same order as the triplet decay constants of TX_DNA_ and Ntl_DNA_ (*k*_TTET_ > *k*_bTTET_ ∼ *k*_TX_, *k*_Ntl_). As a result, an equilibrium-like state is reached on a much slower time scale (Figure S9C).

Lastly, the DNA double strands **TX-Ntl-2** and **TX-2** were examined. Virtually identical TA spectra are observed after excitation (Figure 4A). However, in contrast to the pair with the shorter donor–acceptor distance investigated in Figure 3, kinetic traces of ^3^TX_DNA_ yield essentially identical lifetimes, taking experimental errors into account. Only a minor lifetime decrease from 55.3 ± 0.8 to 55.0 ± 0.9 μs is observed with the introduction of the Ntl C-nucleotide into the DNA double strand (Figure 4B). The high photostability of the DNA samples allowed us to perform several independent sets of measurements, supporting the reliability of the obtained lifetimes (see Section S4.7). Based on this, we calculated an energy transfer constant of (100 ± 400) s^–1^. However, we would like to note that the deviation is higher than the calculated energy transfer constant and should be seen as an upper limit while *k*_TTET_ = 0 s^–1^ sets the lower limit. In any case, at this donor–acceptor distance with four A-T base pairs in-between the donor and the acceptor (17 Å), TTET becomes negligibly small. Given that ^3^TX_DNA_ is virtually not quenched by Ntl in **TX-Ntl-2** on this long time scale which would enable efficient diffusion of large DNA fragments, we can also exclude the possibility of inter-DNA energy transfer as a competing pathway alongside the primary intra-DNA energy migration. In a somewhat related system with a methoxyxanthone C-nucleoside as light absorber, the CPD yields are very similar for double stands with zero or four separating A-T base pairs.^65^ The completely different behavior observed in this study with a drastically more pronounced distance dependence already indicates a Dexter-like instead of a FRET-like energy migration mechanism.

**Figure 4:**
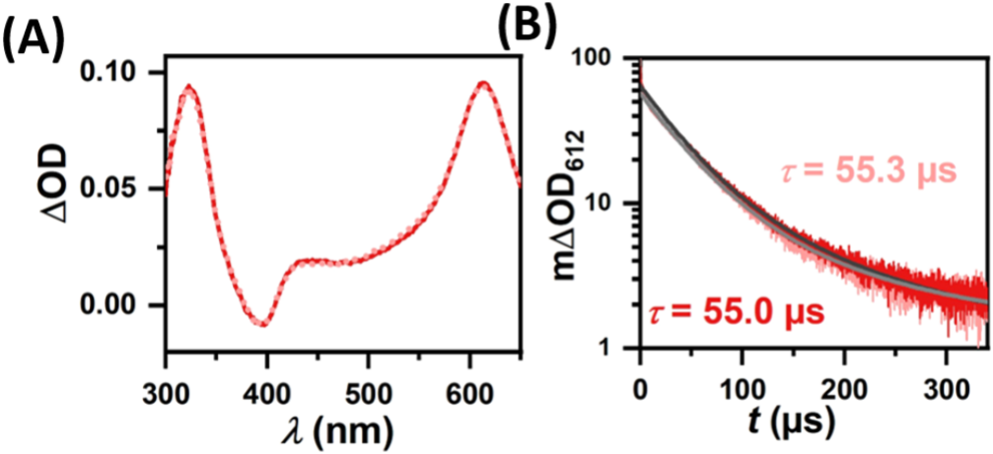
Mechanistic studies of **TX-Ntl-2** (dark red) and **TX-2** (light red) with 355 nm laser pulses. (A) TA spectra recorded 100 ns after excitation. (B) Kinetic TA measurements at 612 nm.

During our experimental work, we observed a secondary process in all TX-containing DNA samples following 355 nm laser excitation, likely associated with the neighboring DNA bases. However, this process is unrelated to the primary focus of this study on triplet energy migration and does not affect the results. A detailed description, along with several control experiments, is provided in the Supporting Information (Section S4.3).

### Distance-Dependence of Triplet–Triplet Energy Transfer

The above-mentioned triplet–triplet energy transfer rates in the three DNA samples with varying donor–acceptor separation were plotted against the respective donor–acceptor distance on a semilogarithmic scale (Figure 5). The distance dependence of electron transfer (ET) and TTET, both governed by exchange-type mechanisms between a donor and an acceptor, is empirically described by the exponential rate equation *k*_DA_ = *k*_0_ **·** exp­(−β **·** *r*_DA_).^20,21,66,67^ Where β is the attenuation factor and it is fundamentally determined by the effective barrier height, Δ*E*_eff_, which inversely correlates with the probability of electron tunneling.^68,69^ In the so-called superexchange mechanism, coupling between donor and acceptor is facilitated by orbitals of bridging units, which greatly surpasses the two-electron exchange integral in magnitude.^70,71^ In a simplified model proposed by Closs et al.,^72^ the attenuation factor for ET is approximately half that of TTET, which can be conceptualized as the simultaneous exchange of two electrons broadly supported by *ab initio* and semiempirical calculations.^73^ However, this assumption does not hold true, when intermediate virtual donor–acceptor CT or bridge-localized excited states are involved in the energy transfer pathways.^19^ In such a scenario, the β value can be lower than the sum of the superexchange constants of electron and hole transfer (see Section S5 for further explanations).^71,74^

**Figure 5:**
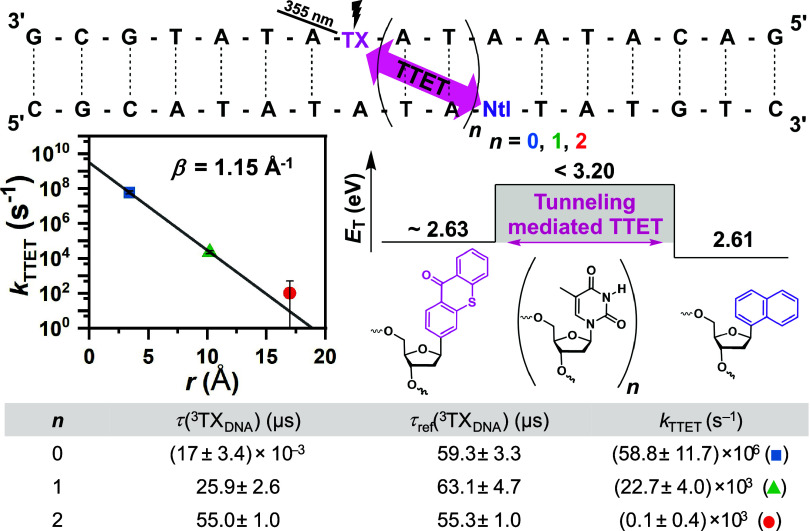
Sequences of DNA double strands **TX-Ntl-*n***, experimentally observed distance dependence of triplet–triplet energy transfer from ^3^TX_DNA_ to Ntl_DNA_, schematic energy diagram of triplet–triplet energy transfer with plausible tunneling mechanism via the thymine triplet state. The table displays the quenched (τ for **TX-Ntl-*n***) and unquenched (τ_ref_ for **TX-*n***) TX triplet lifetimes and the resulting TTET rate constants. See Table S1 for details.

In environments with high barrier heights (Δ*E*_eff_ > 1 eV), such as vacuum or certain solvents, β values typically range from 1.5 to 5 Å^–1^.^74,75^ Molecular bridging significantly enhances the exchange mechanism between donor and acceptor attributed to a reduced Δ*E*_eff_ and low lying virtual bridge states that mediate electronic coupling. For instance, TTET between Ru and Os complexes employing aromatic spacers such as phenyleneethynylene^76,77^ and phenylene^78^ units are typically associated with attenuation factors <1 Å^–1^. If the effective barrier height approaches the available thermal energy, TTET and ET can occur via a hopping mechanism with real intermediate states, significantly extending the transfer range and reducing β to values as low as <0.1 Å^–1^.^79,80^

The DNA double helix presents a distinctive structure with arrays of π-stacked nucleobases separated by 3.4 Å. This configuration favors charge and energy transfer along the bases (through-space) rather than along the sugar–phosphate backbone (through-bond).^81^ Tunneling and hopping mechanisms for charge transfer, particularly hole transfer, have been extensively studied in DNA systems.^81–90^ In contrast, research on TTET within DNA remains limited and is usually based on indirect detection methods.^24,26,30,35^ The handful of studies on this topic are further complicated by the combined effects of Förster- and Dexter-type energy transfer between intercalated donor and acceptor molecules.^30,35^ These interactions obscure a clear understanding of the underlying mechanisms leading to vastly different β values and contradicting interpretations.^31^

Among the free nucleotides thymidine has the lowest triplet state energy (*E*_T_ = 3.2 eV).^40,91,92^ In recent studies, led by one of our groups, the distance dependence of triplet–triplet energy transfer in DNA using high triplet energy donors was thoroughly investigated by detecting the formation of T–T dimers. When sensitizers with *E*_T_ > 3.0 eV where employed, a shallow distance dependence (0.13–0.37 Å^–1^) suggested that a hopping mechanism was operative.^24,26^ The triplet energies of the free nucleotides (≥3.2 eV) cannot support such a hopping mechanism, however stabilizing effects in DNA can significantly lower triplet state energies.^93^ For instance, an average triplet state energy of 2.8 eV has been inferred for thymine in supercoiled circular DNA.^47^ Accurately determining triplet state energies of nucleobases within the DNA stack is challenging, as they are influenced by distinct local environments. Recently, the triplet state energy of thymine in poly-A-T sequences – most relevant to our system – has been calculated at 3.0 eV using a free energy perturbation method that accounts for DNA effects.^94^

Applying the above-mentioned fit function, we obtain an attenuation factor of 1.15 Å^–1^, which is considerably higher than values reported in previous studies of long-range TTET in DNA using triplet sensitizers with *E*_T_ > 3.0 eV^24,26^ and more resembling reports of insulating bridging units in other molecular systems.^68,95^ Hence, a hopping mechanism involving intermediate states of DNA bases^26,34,39^ is unlikely, which is consistent with the absence of triplet state signals from DNA bases in the transient absorption spectra.^40^ Considering the effective barrier height between the triplet donor (∼2.63 eV) and the bridge (calculated at 3.0 eV for poly-A-T sequences) the attenuation factor supports a concerted TTET exchange mechanism, however, the involvement of virtual bridge or CT states cannot be excluded completely (see Section S5 for details).^94^ Based on these findings, we propose that a tunneling mechanism facilitates intra-DNA energy transfer in **TX-Ntl-1** and most likely in **TX-Ntl-2** as well.^70,76,77,96^ The most plausible scenario involves a through-space mediated exchange interaction across the A-T pairs (Figure 5).

## Conclusions

In this study, we have demonstrated that the DNA structure supports triplet energy transfer between thioxanthone (donor) and naphthalene (acceptor), which proceeds via a tunneling mechanism when a direct donor–acceptor orbital overlap is not feasible. Both chromophores were placed as artificial C-nucleotides at defined places in the DNA sequences. Through transient absorption spectroscopy, we provide direct evidence for triplet–triplet energy transfer and determined energy transfer rates for three specific donor–acceptor separations: directly adjacent (3.4 Å), separated by two (10.2 Å) and by four alternating A-T base pairs (17 Å). From these measurements, we calculated an attenuation factor of 1.15 Å^–1^, consistent with related DNA electron transfer studies. Notably, the energy transfer rate observed in the DNA double strand with longest separation between donor and acceptor shows that energy migration becomes negligible at a donor–acceptor separation of four base pairs (17 Å) comparable to molecular donor–acceptor systems with insulating bridging units.^68,95^ This highlights DNA as a relatively poor conductor of triplet energy when hopping is thermodynamically unfeasible. While the triplet state energy of nucleobases is reduced due to π-stacking in DNA, it remains sufficiently high to suppress long-ranged energy migration by UVA-absorbing triplet donors and is likely entirely impractical when using visible light-absorbing sensitizers, whose triplet energies are usually well below 2.7 eV.^45,97^ This serves as an effective protective mechanism for DNA against sunlight-driven sensitization. These findings align with the conclusions of Miranda et al., who established an alert limit for phototoxicity in chemicals at an *E*_T_ ∼ 2.8 eV.^47^

## Supplementary Material



## Data Availability

All experimental data have been provided in the main text and the SI. The data sets shown in the main paper be found under https://doi.org/10.25358/openscience-12363.

## Notes

Materials and methods, experimental details, characterization data, additional steady-state and time-resolved spectroscopic results, raw data sets, and control experiments (PDF)
