# Supplementary material for: Direct Observation of Triplet–Triplet Energy Transfer in DNA between Energy Donor and Acceptor C‑Nucleotides
Source: JACS Au. 2025 May 27;5(6):2770–8. doi: 10.1021/jacsau.5c00364 (PMC12188399; doi:10.1021/jacsau.5c00364)
Supplement: Supplementary file 1 [file au5c00364_si_001.pdf]

Supporting Information for

Direct Observation of Triplet–Triplet Energy Transfer in DNA between Energy Donor and Acceptor C-Nucleotides

Sebastian Häcker [a§], Till J. B. Zähringer [b§], Hans-Achim Wagenknecht [a*], and Christoph Kerzig [b*]

[a] *Institute of Organic Chemistry, Karlsruhe Institute of Technology (KIT), 76131 Karlsruhe, Germany*
[b] *Department of Chemistry, Johannes Gutenberg University Mainz, Duesbergweg 10-14, 55128 Mainz, Germany*

* Email: wagenknecht@kit.de
* Email: ckerzig@uni-mainz.de
§ S.H. and T.J.B.Z. contributed equally to this paper, names ordered alphabetically.

Table of Contents

1. General information 2
2. DNA synthesis and purification 4
3. Synthesis 5
4. Additional spectroscopic measurements 8
   4.1 Triplet-triplet energy transfer of ^{3}TX to Ntl in MeCN 8
   4.2 Measurements with DNA control samples 9
   4.3 Characterization of photoproduct X 9
   4.4 Fluorescence lifetime measurements 13
   4.5 Triplet quenching by oxygen 14
   4.6 TA spectra of **TX-1** and **TX-Ntl-1** 15
   4.7 Kinetic TA traces of **TX-Ntl-2** and **TX-2** 15
   4.8 77 K emission spectra of **TX-Ntl-0** and triplet energy of TX_{DNA} 16
5. Further discussion of the energy transfer mechanism 17
6. Images of ^{1}H, ^{13}C and ^{31}P NMR spectra and of MS analyses 18
7. DNA analyses and melting temperatures 28
8. Images of MS and HPLC analyses of the DNA 30
9. References 32

1. General information

Materials

Unless specifically mentioned, all chemicals used for synthesis or optical spectroscopy were commercially obtained and used as received. The solvents and compounds for optical spectroscopy, together with manufacturer and purity, are as follows: acetonitrile, 99.9%, Fisher Scientific; thioxanthone, 97%, Sigma-Aldrich (purified by recrystallization from toluene/EtOAc prior to use); naphthalene, 97%, Alfa Aesar; deoxyadenosine, >99%, Thermo Scientific; thymidine >99%, Thermo Scientific; NaP_i buffer pH 7: Sodium phosphate monobasic, >98%, Sigma-Aldrich; Sodium phosphate dibasic, >98.5%, Riedel-de Haën; doubly distilled H_2O (Merck Milli-Q Direct 8); ultrapure Millipore MilliQ water with a specific resistance of 18.2 MΩ cm.

Argon from Nippon Gases (5.0) was used for removing dissolved oxygen before all experiments related to optical spectroscopy.

Unmodified oligonucleotides were purchased from Metabion.

NMR spectroscopy

^{1}H NMR (400 MHz), ^{13}C NMR (101 MHz), and ^{31}P NMR (162 MHz) spectra were measured on a Bruker Avance 400. The chemical shifts in the ^{1}H and ^{13}C NMR spectra are reported in parts per million (ppm) relative to tetramethylsilane as an internal standard. The chemical shifts in the ^{31}P NMR spectra are also reported in ppm relative to the deuterated solvent. The coupling constant (J) is given in Hertz (Hz), and the multiplicity of signals are reported as follows: s (singlet), d (doublet), t (triplet), m (multiplet), dt (doublet of triplets), td (triplet of doublets), dd (doublet of doublets), tt (triplet of triplets) and ddd (doublet of doublet of doublets).

Mass Spectrometry

Mass spectrometry was performed on a Finnigan MAT 95 with FAB and EI as ionization methods. ESI mass spectrometry was performed on a Thermo Fisher Scientific Q Exactive (Orbitrap). Oligonucleotides were identified by MALDI mass spectrometry using an AXIMA Confidence spectrometer from Shimadzu. The matrix contained 3-hydroxypicolinic acid (in MeCN/ doubly distilled H_2O 1:1) and diammonium hydrogen citrate (0.44 M in doubly distilled H_2O) in a 9:1 ratio.

HPLC

A Thermo Scientific Dionex Ultimate 3000 HPLC system, equipped with an autosampler, pump module, column oven, multi-diode array detector, fluorescence detector and fraction collector, was used for the semi-preparative purification of oligonucleotides as well as for their analytical characterization. A Supercosil LC 318 column (25 cm x 10 mm, 5 μm) from VDS Optilab was used for the semi-preparative purification. A VDSpher OptiBio Pur 300 S18-SE from VDS Optilab was used for the analytical determination of the oligonucleotides.

Melting temperatures

Melting temperatures of DNA double strands (2.5 μM DNA, 250 mM NaCl, 10 mM $Na\text{-}P_i$ buffer, 10-90 °C, 0.5 °C/min, step width 0.5 °C) were recorded with a Cary 3500 Multicell UV-Vis spectrometer.

Steady-state measurements

Absorption spectra were recorded with a Perkin Elmer LAMBDA 365 UV-Vis spectrophotometer. All spectra were recorded at room temperature (295 ± 3 K) using 10 mm quartz glass cuvettes.

Laser flash photolysis (LFP)

The LP980KS setup from Edinburgh Instruments equipped with an Nd:YAG laser from Quantel (Q-smart 450) was employed for transient absorption and emission spectroscopy. The frequency-tripled (355 nm) output served as the excitation source. The laser pulse duration was ~5 ns and the pulse frequency was 10 Hz. The typical pulse energy used for transient absorption and emission studies was ~20 mJ. A constant laser pulse energy was used within a series of experiments, which was ensured by power measurements before, after and in-between the experiments. The laser power was measured using a pyroelectric detector with attenuator (QE25LP-S-MB-QED-D0) from Gentec-eo combined with the integra software. Detection of transient absorption spectra occurred on an iCCD camera from Andor. Kinetic traces at selected wavelengths were recorded using a photomultiplier tube. The spectroscopic experiments were performed at 293 K using a cuvette holder that allows temperature control. If not stated otherwise the TA spectra were integrated over 100 ns. Longpass filters (385 nm or 400 nm, both from Newport) were used for transient absorption measurements with detection wavelengths exceeding 600 nm to exclude contributions from second-order signals.

77 K Phosphorescence measurements

77 K measurements were carried out with the LP980KS setup. The standard cuvette holder was replaced by a dewar that holds a 4 mm glass tube surrounded by liquid nitrogen.

Time-correlated single photon counting (TCSPC)

The *mini*-τ setup from Edinburgh Instruments (time-correlated single photon counting (TCSPC) technique) equipped with a pulsed laser (EPL-375, excitation at 371.1 nm, pulse width 58.3 ps) was employed for fluorescence lifetime measurements of the DNA double strands at 293 K. Stray light was removed with band pass filters and neutral density filters were used to avoid oversaturation of the detector.

Excited state lifetime determination and TTET rate constants

If not stated otherwise, a biexponential fit function was used to determine the weighted average lifetime of the fluorescence and transient triplet-excited states. The kinetic transient absorption spectra of **TX-Ntl-n** and **TX-n** (n = 0, 1, 2) where fitted after 4 µs after laser pulse excitation in order to exclude the secondary process (see Section S4.3 for details).

Triplet energy transfer rates, k_{TTET}, within the DNA double strands between TX and Ntl were determined by the following equation. Where τ_{obs} is the measured lifetime of the DNA double strand (**TX-Ntl-n**, n = 0, 1, 2) and τ_{ref} is the reference triplet state lifetime of TX in the absence of Ntl (**TX-n**, n = 0, 1, 2),

$$k_{\mathrm{TTET}} = \frac{1}{\tau_{\mathrm{obs}}} - \frac{1}{\tau_{\mathrm{ref}}} = k_{\mathrm{obs}} - k_{\mathrm{ref}} \quad (1)$$

Table S1: Triplet state decay constants k_{ref} of **TX-n** (n= 0, 1, 2), triplet state decay constants k_{obs} of **TX-Ntl-n** (n= 0, 1, 2) and calculated TTET rate constants k_{TTET}.

Sample	k_{ref} (s^{-1})	k_{obs} (s^{-1})	k_{TTET} (s^{-1})
**TX-Ntl-0**	$(16.9 \pm 0.9) \times 10^3$	$(58.8 \pm 11.7) \times 10^6$ [a]	$(58.8 \pm 11.7) \times 10^6$
**TX-Ntl-1**	$(15.9 \pm 1.1) \times 10^3$	$(38.6 \pm 3.9) \times 10^3$ [b]	$(22.7 \pm 4.0) \times 10^3$
**TX-Ntl-2**	$(18.1 \pm 0.3) \times 10^3$	$(18.2 \pm 0.3) \times 10^3$	$(0.1 \pm 0.4) \times 10^3$

[a] Rate determined from the faster component of the biexponential fit function in Figure 2C (main text). Estimated relative error 20%. [b] Rate obtained by fitting the curve in the 4-14 µs range monoexponentially shown in Figure 3B (main text). Estimated relative error 10%.

2. DNA synthesis and purification

Reagents and controlled-pore glass (CPG) (1 µmol) were purchased from ABI and GlenResearch. Oligonucleotide synthesis was carried out under an argon atmosphere using an H-6 DNA/RNA synthesizer from K&A Laborgeräte. The coupling protocol was modified for the incorporation of thioxanthone- and naphthalene-phosphoramidites (Table S2). The modified oligonucleotides were synthesized as trityl-on oligonucleotides and pre-purified using Glen-Pak DNA Purification Cartidges before purification by semi-preparative HPLC with the following conditions: mobile phase A = NH_4OAc buffer (50 mM, pH 7), mobile phase B = acetonitrile, flow rate 2.5 mL/min, UV/Vis detection at 260, 280 and 310/385 nm. After purification, the oligonucleotides were lyophilized, and their concentrations were determined by absorbance at 260 nm using a Nanodrop ND-1000 spectrophotometer. Analytical characterization of purified oligonucleotides was performed via RP-HPLC under the following conditions: mobile phase A = NH_4OAc buffer (50 mM, pH 7), mobile phase B = acetonitrile (gradient: 1-45% B), flow rate = 1.0 mL/min, UV/Vis detection at 260, 280 and 310/385 nm.

Table S2: Coupling protocol of thioxanthone- and naphthalene-phosphoramidite.

	**Time [0.1 s]**	**Source**	**Mixed**	**Destin**	**S.Col.Ptr.**	**Lag time [s]**	**Branch**
BRANCH							
**1**	4	TET		COL	ON		
**2**	6	AMD	TET	COL			
**3**					ON		
**4**						99	
**5**						99	
**6**						99	
**7**						99	
**8**						99	
**9**						99	
**10**						99	
**11**						99	
**12**	6	AMD	TET	COL	ON		
**13**					ON		
**14**						99	
**15**						99	
**16**						99	
**17**						99	
**18**						99	
**19**						99	
**20**						99	
**21**						99	
**22**	4	TET		COL	ON		
**23**					ON		
**24**	20	ACN		M_W			
**25**	20	GAS		M_W			

3. Synthesis

Synthesis of **2**

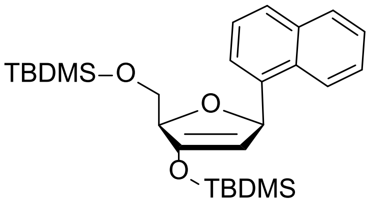

2a

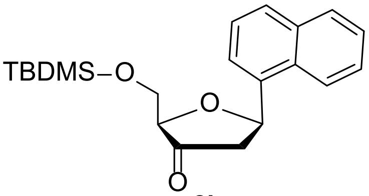

2b

In an argon-flushed flask 1-bromonaphthalene (1.00 eq.), Glykal **1** (1.00 eq.) and triethylamine (2.15 eq.) were dissolved in dimethylformamide, stirred at room temperature for 10 min and degassed using freeze-pump-thaw method. Subsequently $Pd_2(dba)_3$ (0.05 eq.) and Q-Phos (0.20 eq.) were added and the reaction mixture was stirred at 80 °C for 72 h. The solvent was removed under reduced pressure and the crude product was purified by column chromatography (SiO_2, gradient cyclohexane/EtOAc 10:1 → 3:1) to obtain **2a** (44%) and **2b** (35%) as a colorless solid.

2a:

^{1}H NMR (400 MHz, $CDCl_3$) δ 8.15 – 8.10 (m, 1H), 7.88 – 7.84 (m, 1H), 7.81 – 7.74 (m, 2H), 7.54 – 7.42 (m, 3H), 6.53 (dd, *J* = 3.8, 1.6 Hz, 1H), 5.03 (t, *J* = 1.8 Hz, 1H), 4.69 (tt, *J* = 4.3, 2.2 Hz, 1H), 3.91 (dd, *J* = 11.2, 2.3 Hz, 1H), 3.76 (dd, *J* = 11.2, 4.6 Hz, 1H), 0.96 (s, 9H), 0.83 (s, 9H), 0.23 (d, *J* = 17.4 Hz, 6H), –0.07 (d, *J* = 3.8 Hz, 6H).

^{13}C NMR (101 MHz, $CDCl_3$) δ 151.30, 138.87, 133.70, 131.19, 128.75, 127.91, 126.03, 125.73, 125.45, 124.19, 123.45, 101.34, 84.37, 80.93, 64.73, 27.07, 26.12, 25.73, 18.64, 18.22, –4.69, –4.73, –5.26, –5.31.

HR-MS (ESI, $[M+H]^+$) m/z: found: 471.2744; calc.: 471.2745.

2b:

^{1}H NMR (400 MHz, $CDCl_3$) δ 8.04 – 7.99 (m, 1H), 7.94 – 7.87 (m, 2H), 7.83 (d, *J* = 8.2 Hz, 1H), 7.56 – 7.47 (m, 3H), 5.89 (dd, *J* = 11.2, 5.7 Hz, 1H), 4.16 (t, *J* = 2.6 Hz, 1H), 4.10 – 4.01 (m, 2H), 3.04 (dd, *J* = 17.7, 5.7 Hz, 1H), 2.55 (dd, *J* = 17.7, 11.2 Hz, 1H), 0.88 (s, 9H), 0.12 (s, 3H), 0.08 (s, 3H).

^{13}C NMR (101 MHz, $CDCl_3$) δ 129.12, 128.64, 126.47, 125.97, 125.82, 125.77, 123.28, 123.03, 120.47, 119.58, 110.71, 82.94, 75.10, 62.68, 45.92, 25.97, –5.24, –5.47.

HR-MS (ESI, $[M+H]^+$) m/z: found: 357.1879; calc.: 357.1880.

Synthesis of 3

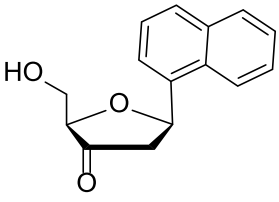

3

In an argon-flushed flask **2** (1.00 eq.) was dissolved in dry THF. The mixture was cooled down to 0 °C, $Et_3N\cdot 3HF$ (8.00 eq.) was added and stirred at room temperature overnight. The solvent was removed under reduced pressure and the crude product was purified by column chromatography (SiO_2, gradient CH_2Cl_2 → CH_2Cl_2/MeOH 10:1) to obtain **3** as a colorless solid (60%).

^{1}H NMR (400 MHz, $CDCl_3$) δ 8.03 – 7.97 (m, 1H), 7.91 – 7.83 (m, 2H), 7.75 (d, *J* = 7.1 Hz, 1H), 7.58 – 7.49 (m, 3H), 5.90 (dd, *J* = 11.1, 5.7 Hz, 1H), 4.17 (t, *J* = 3.5 Hz, 1H), 4.05 – 3.97 (m, 2H), 3.11 – 3.03 (m, 1H), 2.71 – 2.61 (m, 1H).

^{13}C NMR (101 MHz, $CDCl_3$) δ 213.93, 135.31, 133.88, 130.66, 129.13, 129.07, 126.64, 126.03, 125.61, 123.09, 122.79, 82.43, 75.08, 61.65, 44.79.

HR-MS (ESI, $[M+H]^+$) m/z: found: 243.1015; calc.: 243.1016.

Synthesis of 4

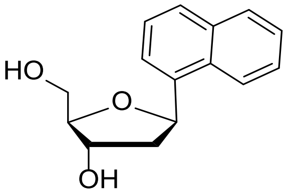

4

In an argon-flushed flask **3** (1.00 eq) was dissolved in AcOH/MeCN (1:3) and sodium triacetoxyborohydride (8.00 eq) was added at 0 °C. The reaction mixture was stirred at room temperature for 40 min and quenched afterwards with the same volume EtOH/H_2O (1:1). The solvent was removed under reduced pressure and the crude product was purified by column chromatography (SiO_2, gradient CH_2Cl_2→ CH_2Cl_2/MeOH 10:1) to obtain **4** as a colorless solid in a yield of 80%.

^{1}H NMR (400 MHz, $CDCl_3$) δ 8.09 – 8.01 (m, 1H), 7.87 (dd, *J* = 7.9, 1.7 Hz, 1H), 7.79 (d, *J* = 8.2 Hz, 1H), 7.65 (dt, *J* = 7.3, 1.1 Hz, 1H), 7.56 – 7.45 (m, 3H), 5.90 (dd, *J* = 10.1, 5.7 Hz, 1H), 4.50 (dt, *J* = 6.1, 2.7 Hz, 1H), 4.13 (td, *J* = 4.5, 3.4 Hz, 1H), 3.95 – 3.79 (m, 2H), 2.51 (ddd, *J* = 13.3, 5.8, 2.3 Hz, 1H), 2.15 (ddd, *J* = 13.3, 10.1, 6.5 Hz, 1H).

^{13}C NMR (101 MHz, $CDCl_3$) δ 137.08, 133.83, 130.81, 128.94, 128.26, 126.27, 125.81, 125.62, 123.48, 122.02, 87.03, 73.93, 63.60, 43.30.

HR-MS (ESI, $[M]^+$) m/z: found: 244.1094; calc.: 244.1094.

Synthesis of 5

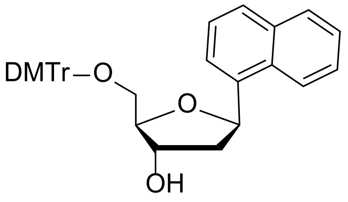

5

In an argon-flushed flask **4** (1.00 eq.) was dissolved in dry pyridine. 4,4`-Dimethoxytriphenylmethylchlorid (1.50 eq.) was gradually added to the reaction mixture and stirred at room temperature overnight. The solvent was removed under reduced pressure and the crude product was purified by column chromatography (SiO_2, gradient CH_2Cl_2→ CH_2Cl_2/acetone 20:1) to obtain **5** as a colorless solid in a yield of 63%.

^{1}H NMR (400 MHz, $CDCl_3$) δ 7.99 – 7.91 (m, 1H), 7.80 – 7.75 (m, 1H), 7.71 – 7.52 (m, 2H), 7.47 – 7.27 (m, 4H), 7.23 – 7.12 (m, 5H), 7.10 – 7.04 (m, 3H), 6.73 (d, *J* = 8.9 Hz, 4H), 5.79 (dt, *J* = 9.5, 6.4 Hz, 1H), 4.39 (ddd, *J* = 7.4, 5.3, 2.9 Hz, 1H), 4.08 – 3.98 (m, 1H), 3.77 (ddd, *J* = 31.1, 11.4, 4.3 Hz, 2H), 2.48 – 2.33 (m, 1H), 2.13 – 1.98 (m, 1H).

^{13}C NMR (101 MHz, $CDCl_3$) δ 158.78, 139.60, 130.27, 129.28, 128.93, 128.48, 128.36, 128.24, 128.00, 127.91, 127.23, 126.25, 126.09, 125.79, 125.62, 123.48, 122.01, 113.32, 87.03, 73.95, 63.61, 55.40, 43.33.

HR-MS (EI, $[M]^+$) m/z: found: 546.2399; calc.: 546.2401..

Synthesis of 6

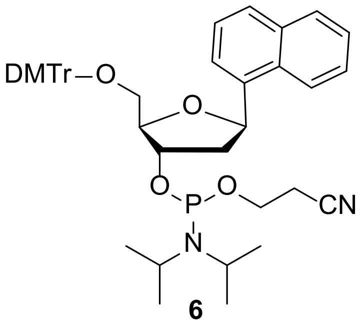

6

In an argon-flushed flask **5** (1.00 eq.) was dissolved in abs. CH_2Cl_2. N,N-Diisopropylethylamine (3.50 eq) was added to the reaction mixture at room temperature. Subsequently, 2-Cyanoethyl N,N-diisopropylchlorophosphoramidite (1.50 eq.) was added and the mixture stirred for 3 h. The crude product was purified by column chromatography (SiO_2, CH_2Cl_2/acetone 50:1 + 0,1% Et_3N) to obtain **6** as a colorless solid in a yield of 98%.

^{1}H NMR (400 MHz, $CDCl_3$) δ 8.12 – 8.05 (m, 1H), 7.91 – 7.84 (m, 1H), 7.83 – 7.74 (m, 2H), 7.53 – 7.42 (m, 5H), 7.39 (ddd, *J* = 8.8, 4.3, 1.9 Hz, 4H), 7.31 – 7.27 (m, 1H), 7.25 – 7.17 (m, 2H), 6.85 – 6.77 (m, 4H), 5.88 (dt, *J* = 10.3, 5.3 Hz, 1H), 4.64 – 4.53 (m, 1H), 4.35 – 4.29 (m, 1H), 3.92 – 3.80 (m, 1H), 3.80 – 3.77 (m, 6H), 3.77 – 3.54 (m, 3H), 3.46 – 3.31 (m, 2H), 2.72 – 2.52 (m, 2H), 2.52 – 2.44 (m, 1H), 2.21 – 2.08 (m, 1H), 1.30 – 1.15 (m, 9H), 1.10 (d, *J* = 6.8 Hz, 3H).

^{31}P NMR (162 MHz, $CDCl_3$) δ 148.4, 147.9.

HR-MS (ESI, $[M+H]^+$) m/z: found: 747.3550; calc.: 747.3558.

4. Additional spectroscopic measurements

4.1 Triplet-triplet energy transfer of ^{3}TX to Ntl in MeCN

To serve as a reference for the DNA samples, we characterized a solution of TX both in the presence and absence of 1 mM Ntl in deaerated acetonitrile (MeCN) using transient absorption spectroscopy. It is important to note that the triplet state lifetime of both TX and Ntl is significantly influenced by excitation intensity and excited state concentration, which can lead to self-quenching and annihilation.[1–3] Upon excitation, TX nearly quantitatively and instantaneously forms the triplet state, exhibiting characteristic maxima at 320 nm and 625 nm, along with a ground-state bleach (GSB) at 380 nm in the transient absorption spectrum (Figure S1A, red spectrum) with a measured lifetime of 23.0 µs (Figure S1B).[4] In the presence of 1 mM Ntl (Figure S1C, purple spectrum), ^{3}TX is quantitatively (>99%) quenched (τ_{obs} = 19 ns) according to excited state quenching dynamics,[5] and ^{3}Ntl is formed with a maximum at 410 nm (Figure S1A). The triplet state lifetime of Ntl was measured to be 15.0 µs (Figure S1B) under our conditions. The triplet energy of TX is 2.75 eV,[4] which is 0.12 eV higher than that of Ntl (2.63 eV);[5] this thermodynamic driving force is sufficient for an approximately diffusion-controlled (k_{TTET} ~ 10^{10} M^{-1} s^{-1}) energy transfer rate constant in line with reported energy transfer rate constants for this donor-acceptor system.[6,7]

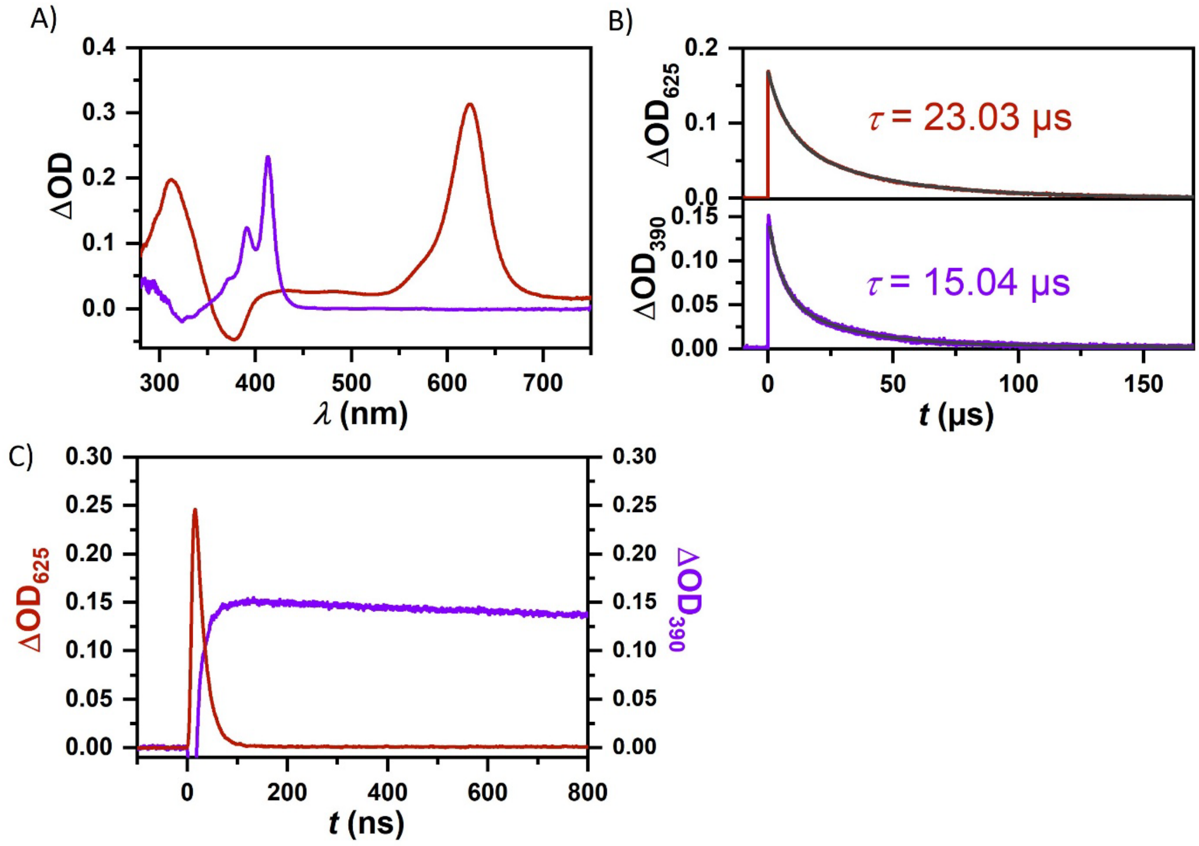

Figure S1: Transient absorption spectra of 20 µM TX in the absence (red) and in the presence of 1 mM Ntl (purple) in deaerated MeCN excited by 355 nm laser pulses of ~5 ns duration (20 mJ per pulse). A) TA spectra recorded 600 ns after excitation. B) Top panel: Time-resolved absorption of ^{3}TX (in the absence of Ntl). Bottom panel: Time-resolved absorption of ^{3}Ntl. C) Simultaneous deactivation of ^{3}TX (625 nm, red) and formation of ^{3}Ntl (390 nm, purple) on a nanosecond timescale.

4.2 Measurements with DNA control samples

To exclude direct excitation of the DNA double strands, two control samples were investigated by LFP with 355 nm laser pulses. The sample **Control** lacked both TX and Ntl, whereas the **Ntl-Control** contained only Ntl, with TX replaced by the initial thymine base (Figure S2A). TA spectra of the control samples in deaerated aqueous solution (250 mM NaCl, 10 mM Na-P_i-buffer) recorded 100 ns after the initial laser pulse did not show the formation of any transient species (Figure S2B and S2C).

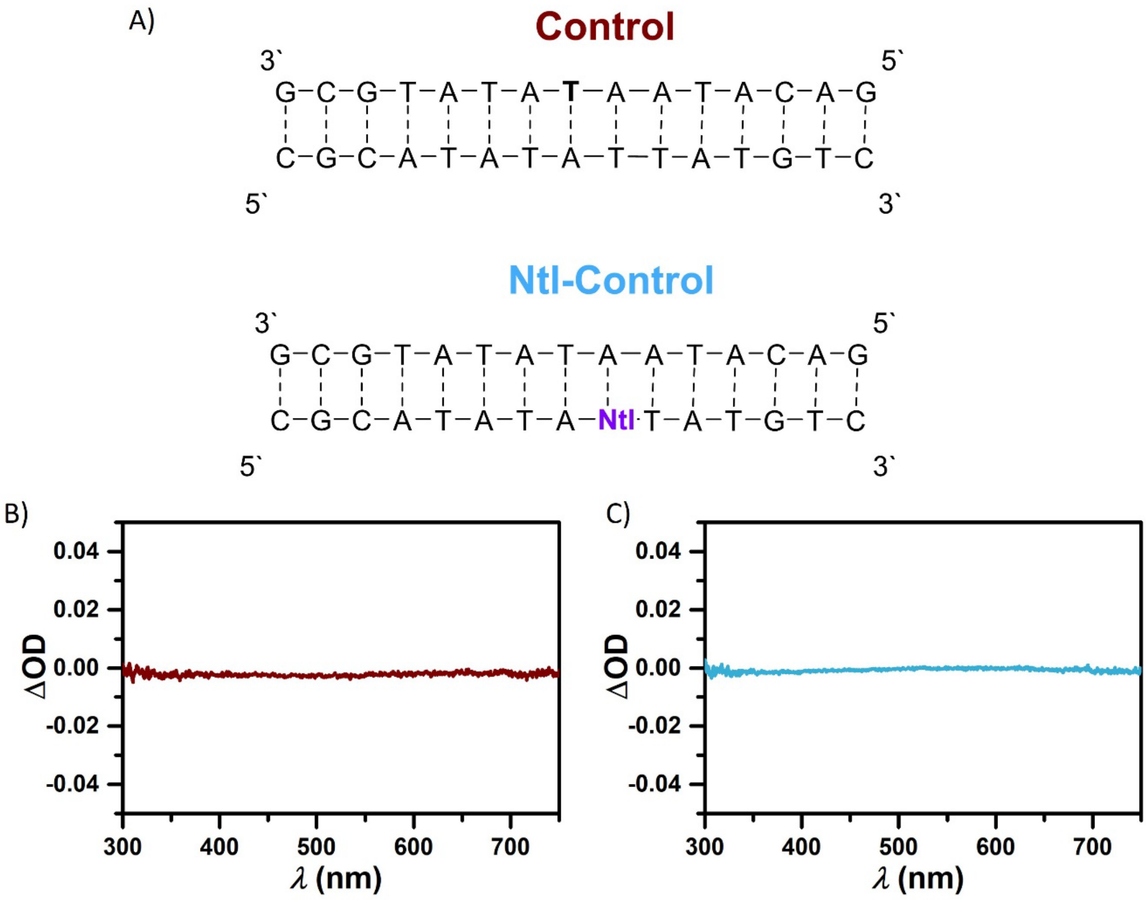

Figure S2: A) Structures of control DNA double strand samples. TA spectra of B) **Control** and C) **Ntl-Control** recorded in deaerated aqueous solution (250 mM NaCl, 10 mM Na-Pi-buffer) 100 ns after 355 nm laser pulses.

4.3 Characterization of photoproduct X

Apart from the triplet energy transfer between TX and Ntl, a secondary independent process was identified. This phenomenon was noted for all DNA samples containing TX and is for example shown for **TX-2** (Figure S3), although essentially identical observations were made for the other TX-containing samples.

Directly after excitation with 355 nm laser pulses an additional transient species was observed, exhibiting a broad absorption peak centered at 610 nm (Figure S3A), with a much faster decay than that of the TX triplet state (Figure S3B, top panel). Furthermore, this decay is accompanied by the formation of another transient species at 525 nm referred to as photoproduct X (Figure S3A and Figure S3B, bottom panel). The decay of X at 525 nm is superimposed by ^{3}TX, and our results indicate that it does not significantly absorb at 612 nm (Figure S3C). However, when compared to the kinetic traces at 612 nm we observe a similar lifetime to ^{3}TX of the photoproduct indicating that the process is reversible. We note that this process was not only observed in all samples but is also independent of the presence or absence of Ntl in the counter strand, albeit to different degrees (Figure S3D).

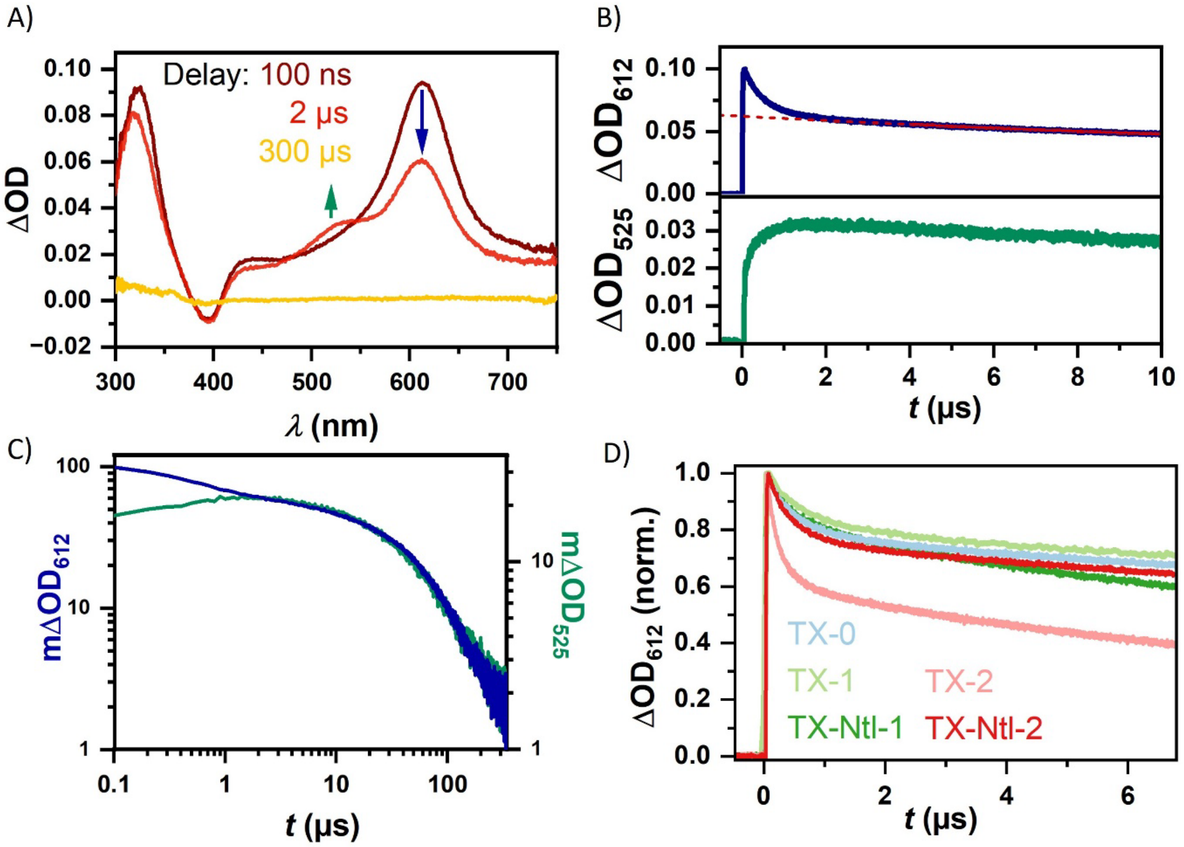

Figure S3: TA spectra of **TX-2** in deaerated aqueous solution (250 mM NaCl, 10 mM Na-P$_i$ buffer). A) Spectra recorded 100 ns, 2 µs and 300 µs after excitation. B) Kinetic measurements at 612 nm (top panel, with monoexponential fit function of the ^{3}TX decay (red line), fitted 4 µs after excitation and extrapolated to the pre-fitting region) and at 525 nm (bottom panel). C) Kinetic measurements at 612 nm and at 525 nm for a comparison of the signals of the unexpected species on a double-logarithmic scale. D) Normalized kinetic traces at 612 nm of several DNA samples.

In contrast, only baseline levels are detected in the absence of TX following 355 nm laser pulses (see Section S4.2). To explore the novel species further the single-stranded DNA containing TX (**ssTX-0**) was examined. Exciting this sample at 355 nm produced results similar to those observed previously. Although the triplet state lifetime of TX is shorter in the single-stranded sample, we still see the formation of the secondary process (Figure S4). Based on these measurements, we conclude that the signals do not stem from denatured double-stranded DNA or interstrand crosslinking following the hydrogen abstraction reaction but instead originate from the strand containing the TX C-nucleotide.

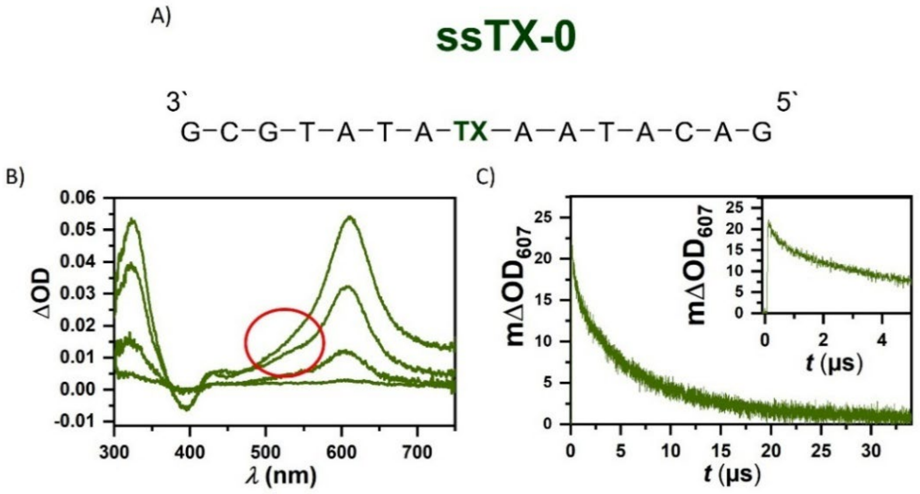

Figure S4: A) Structure of single strand sample **ssTX-0**. B) TA spectra of 90 μM single strand sample in deaerated aqueous solution (250 mM NaCl, 10 mM Na-P$_i$ buffer) recorded 100 ns, 1 μs, 5 μs and 25 μs after 355 nm excitation. The red circle indicates the photoproduct. C) Kinetic measurements at 607 nm. Inset: Post-pulse signals (0-5 μs) on enlarged scale.

It was hypothesized that the secondary process could be a result of a two-photon absorption process, given that photoionization was observed for several chromophores in polar media upon 355 nm ns-laser excitation.[8–10] Hence, we studied the laser excitation intensity dependence of the sample **TX-2** by TA spectroscopy at different time delays and laser intensities ranging from 2.7 mJ to 58 mJ (Figure S5A and B). Selected wavelengths were plotted against the laser intensity (Figure S5C). However, the formation of X at 525 nm (2 μs delay), the broad signal at 612 nm (100 ns delay) and the transient absorption of ^{3}TX at 612 nm (2 μs delay) do not show quadratic behavior, which would be indicative of a two-photon process. The difference transient absorption spectrum of **TX-2** recorded at 100 ns and 2 μs (adjusted for the decay of ^{3}TX) is shown in Figure S5D. The negative signal at 525 nm corresponds to the photoproduct X. The difference spectrum shows two maxima at 320 nm and 612 nm as well as a minimum at 380 nm.

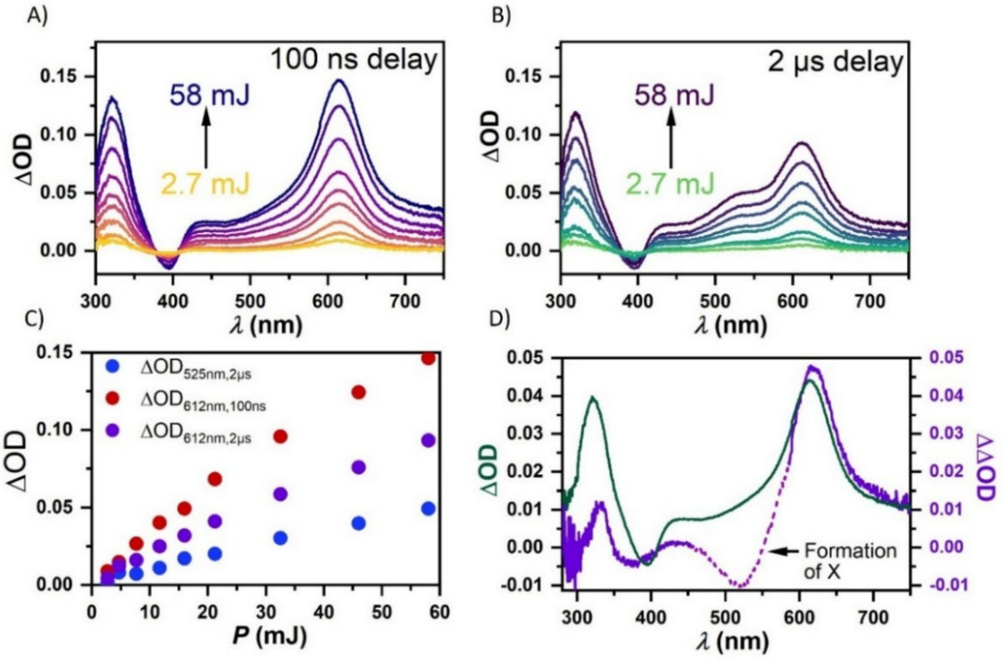

Figure S5: Laser intensity dependent measurements of **TX-2** with excitation energies ranging from 2.7 mJ to 58 mJ. A) TA spectra recorded A) 100 ns and B) 2 μs after excitation. C) Transient absorption signals at selected wavelengths plotted against the laser intensity. D) TA spectrum recorded after 100 ns (green) and difference TA spectrum of 100 ns delay and 2 μs delay (purple), adjusted for the decay of ^{3}TX (compare Figure S3B), the formation of photoproduct X is highlighted by dashed lines.

The close similarity between the difference spectrum in Figure S5D and the TA spectrum of $^3TX_{DNA}$ itself led us to investigate the influence of nearby nucleotides on TX. An Ar-saturated solution of TX in a 4:1 (v/v) mixture of H_2O and MeCN was prepared and examined with and without 100 mM thymidine or deoxyadenosine (Figure S6). The steady-state absorption spectrum of TX was already altered by the addition of 100 mM deoxyadenosine, a change that cannot be explained by the absorption of deoxyadenosine itself (λ_{onset} < 325 nm, Figure S6A). The absorption maximum at 386 nm is slightly red-shifted and its intensity is reduced, while the absorption below 350 nm is increased.

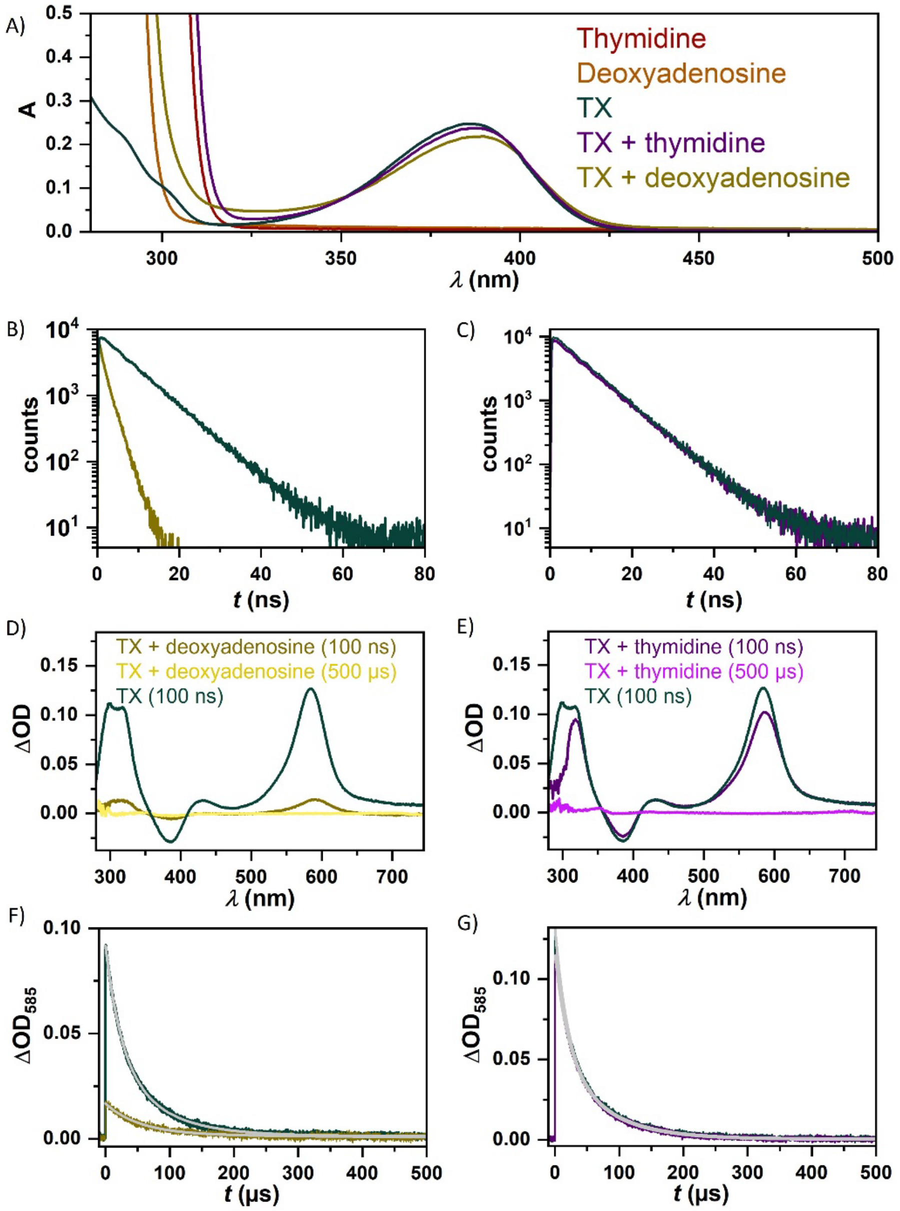

Figure S6: Quenching experiments of 5 µM TX (dark green) with 100 mM of the nucleotides thymidine (violet) and deoxyadenosine (dark yellow) in Ar-saturated H_2O/MeCN (v/v 4:1). A) Absorption spectra of the compounds. B) Time-resolved emission of TX in the presence and absence of deoxyadenosine (same color code as in A). C) Time-resolved emission of TX in the presence and absence of thymidine. D) TA Spectra of TX in the absence (delay: 100 ns) and presence of deoxyadenosine (delay: 100 ns and 500 µs). D) TA Spectra of TX in the absence (delay: 100 ns) and presence of thymidine (delay: 100 ns and 500 µs). F) Time-resolved transient absorption at 585 nm in the presence and absence of deoxyadenosine. G) Time-resolved transient absorption at 585 nm in the presence and absence of thymidine.

This effect was not observed to the same extent when thymidine was added, suggesting that the ribose moiety is not responsible for the change. Fluorescence quenching of TX was only observed with the addition of deoxyadenosine (Figure S6B and C). A reduction in triplet state formation of TX in the presence of deoxyadenosine was noted, likely due to singlet state quenching (Figure S6D). Under the

same conditions, nearly identical triplet absorption spectra of TX were observed with or without thymidine (Figure S6E). These findings appear contradictory, as the excited singlet state of adenosine (> 4 eV)[11] should not be accessible via energy transfer from singlet-excited TX (3.3 eV)[12]. Electron or hydrogen atom transfer reactions can also be excluded (or they are non-productive), as no thioxanthone-derived photoproducts were detected after excitation (Figure S6D and S6E). We propose the formation of a five-membered electron donor-acceptor ring between the carbonyl moiety of thioxanthone and the adenine moiety, as has been suggested for acetone quenching by adenine.[13] The partial protonation of the carbonyl group could explain the red shift in the ground-state absorption and the acceleration of excited singlet state deactivation.[14] An emission shift due to singlet-exciplex formation could not be observed with the addition of adenosine.[15]

Based on these control experiments, it is reasonable to conclude that the adenosine nucleotide adjacent to the thioxanthone C-nucleotide (twice) in the DNA structures may influence the excited state properties of thioxanthone, as observed in Figure S3. However, we note that this interaction between thioxanthone and adenosine occurs consistently across all samples with almost identical kinetics. Therefore, as a result of all control and reference experiments carried out by us, our primary investigation of triplet-triplet energy transfer between $^3TX_{DNA}$ and Ntl_{DNA} is not negatively affected by this interaction.

4.4 Fluorescence lifetime measurements

The fluorescence lifetime of the samples **TX-Ntl-0**, **TX-0**, **TX-Ntl-1**, **TX-1**, **TX-Ntl-2** and **TX-2** were measured by TCSPC using a pulsed laser at 371.1 nm (Figure S7). Using a biexponential fit function we determined τ_1, τ_2 and the amplitude weighted average lifetime τ_{avg} as displayed in Table S3.

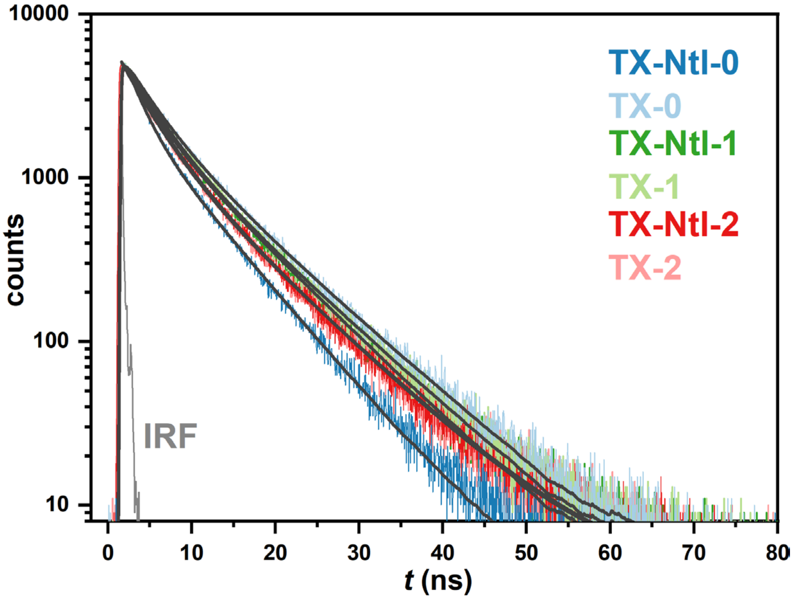

Figure S7: Time-resolved fluorescence measurements of **TX-Ntl-0**, **TX-0**, **TX-Ntl-1**, **TX-1**, **TX-Ntl-2** and **TX-2** in deaerated aqueous solution (250 mM NaCl, 10 mM Na-Pi buffer) upon pulsed laser excitation (371.1 nm) fitted with a biexponential decay function. Lifetimes are given in Table S3. Detection occurred between 425 and 475 nm using a suitable optical filter.

In comparison to the fluorescence lifetime of TX in organic solvents like acetonitrile (MeCN), which is 70 ps, the fluorescence lifetime of TX incorporated into double-stranded DNA samples is over two orders of magnitude longer but comparable to the lifetimes that we obtained in H_2O/MeCN (Figure S6B). This extended lifetime is anticipated when polar protic solvents are utilized. For instance, in methanol (MeOH), the lifetime of TX increases to 2.55 ns, and in a mixture of acetonitrile and water (v/v 3:2), it reaches 4.9 ns.[14,16]

Table S3: Fluorescence lifetimes (τ_1, τ_2, τ_{avg}) of DNA samples **TX-Ntl-0**, **TX-0**, **TX-Ntl-1**, **TX-1**, **TX-Ntl-2** and **TX-2** determined using a biexponential decay function following TCSPC measurements with a pulsed laser at 371.1 nm.

Sample	τ_1 (ns)	τ_2 (ns)	τ_{avg} (ns)
**TX-Ntl-0**	2.07	7.22	4.31
**TX-0**	3.66	9.53	6.40
**TX-Ntl-1**	2.82	8.58	5.66
**TX-1**	3.33	9.21	5.92
**TX-Ntl-2**	3.50	9.19	5.59
**TX-2**	3.35	9.35	5.52

4.5 Triplet quenching by oxygen

In an air-saturated solution, the lifetime of ^{3}TX (**TX-0**) decreases from 59.3 µs to 29.9 µs due to the presence of oxygen, which acts as a triplet state quencher (Figure S8). Taking the high ionic strength into account, the O_2 solubility (c = 0.29 mM in neat water at 20°C)[5] is lower by ~10 % (i.e., 0.261 mM).[17] We estimate a quenching rate constant of ~ $6.4 \cdot 10^7$ M^{-1} s^{-1}, using the observed lifetimes and the above-mentioned oxygen concentration. This is roughly two orders of magnitude lower than diffusion-based quenching in water. We propose that the DNA backbone provides significant shielding to the C-nucleotides of TX and Ntl, effectively preventing inter-DNA energy transfer, which would be highly inefficient at the 90 µM concentration of the DNA samples and could not account for the observed energy transfer rate in **TX-Ntl-1**.

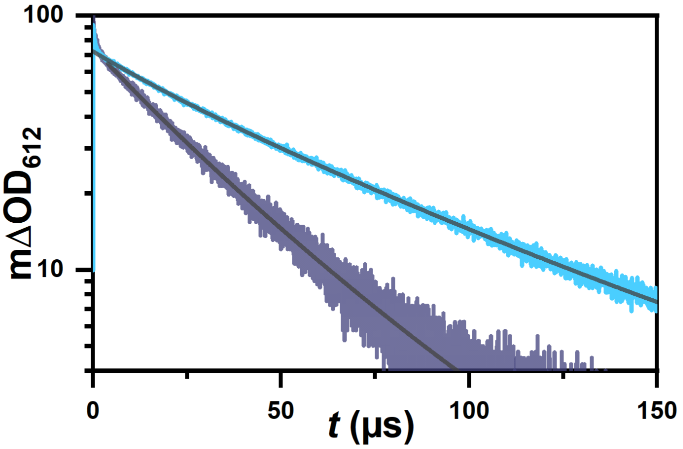

Figure S8: Time-resolved TA traces of **TX-0** in Ar-saturated (blue) and air-saturated (violet) aqueous solution (250 mM NaCl, 10 mM Na-P$_i$ buffer).

4.6 TA spectra of **TX-1** and **TX-Ntl-1**

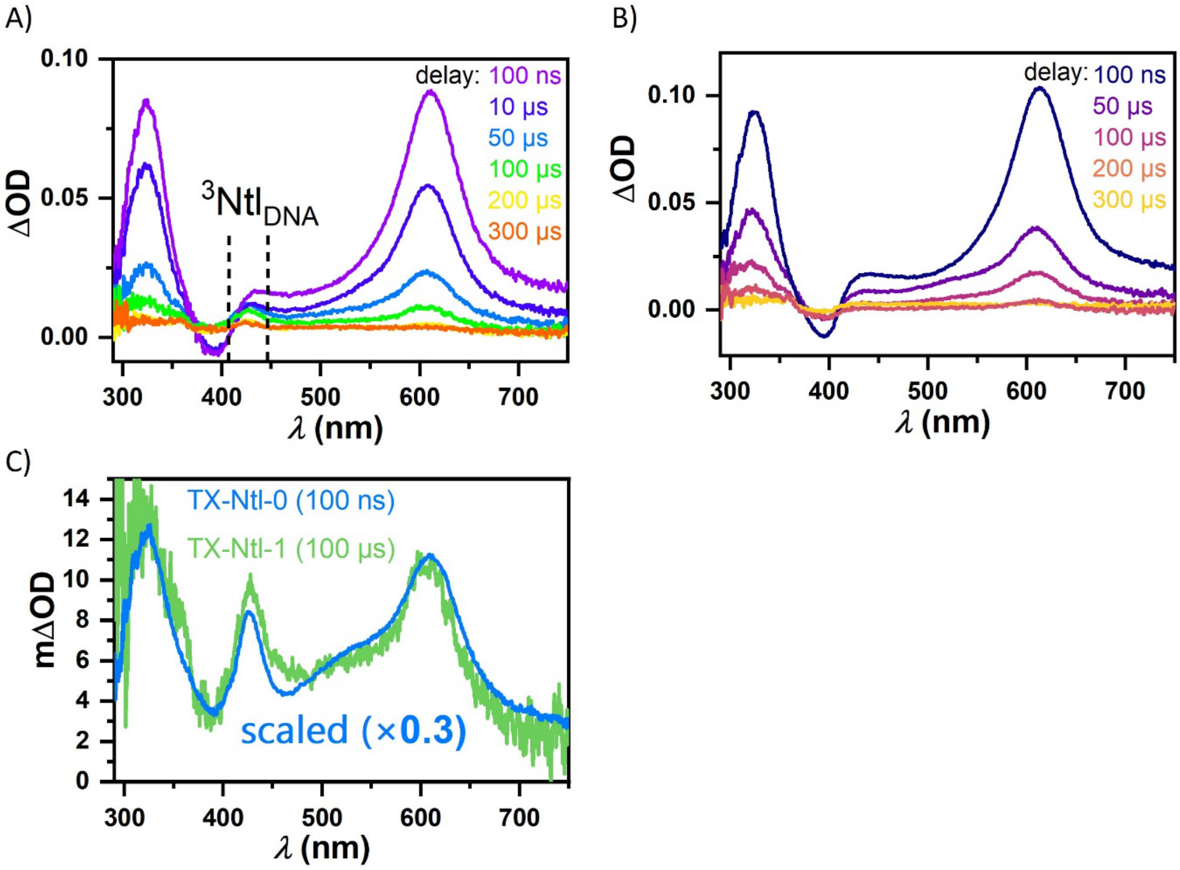

Figure S9: TA spectra of A) **TX-Ntl-1** and B) **TX-1** in deaerated aqueous solution (250 mM NaCl, 10 mM Na-P$_i$ buffer) recorded after different time delays. C) Direct comparison of TA spectra of **TX-Ntl-0** recorded after 100 ns (scaled) and of **TX-Ntl-1** after 100 µs.

4.7 Kinetic TA traces of **TX-Ntl-2** and **TX-2**

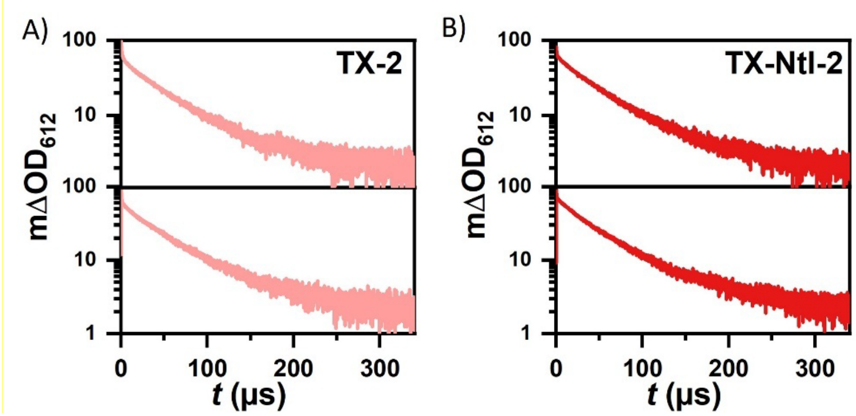

Figure S10: Kinetic TA traces of **TX-2** (A)) and **TX-Ntl-2** (B)) in deaerated aqueous solution (250 mM NaCl, 10 mM Na-P$_i$ buffer), recorded independently. Top panel: initial measurement; Bottom panel: measured after ~ 24 h. Each measurement comprises 40 individual measurements.

Two independent sets of TX$_{DNA}$ lifetime measurements for **TX-2** and **TX-Ntl-2** were conducted on separate days. The consistency between these measurements underscores the high photostability of the DNA samples, supporting the reliability of the obtained lifetimes. Final values were calculated by averaging the results from both sets.

4.8 77 K emission spectra of **TX-Ntl-0** and triplet energy of TX_{DNA}

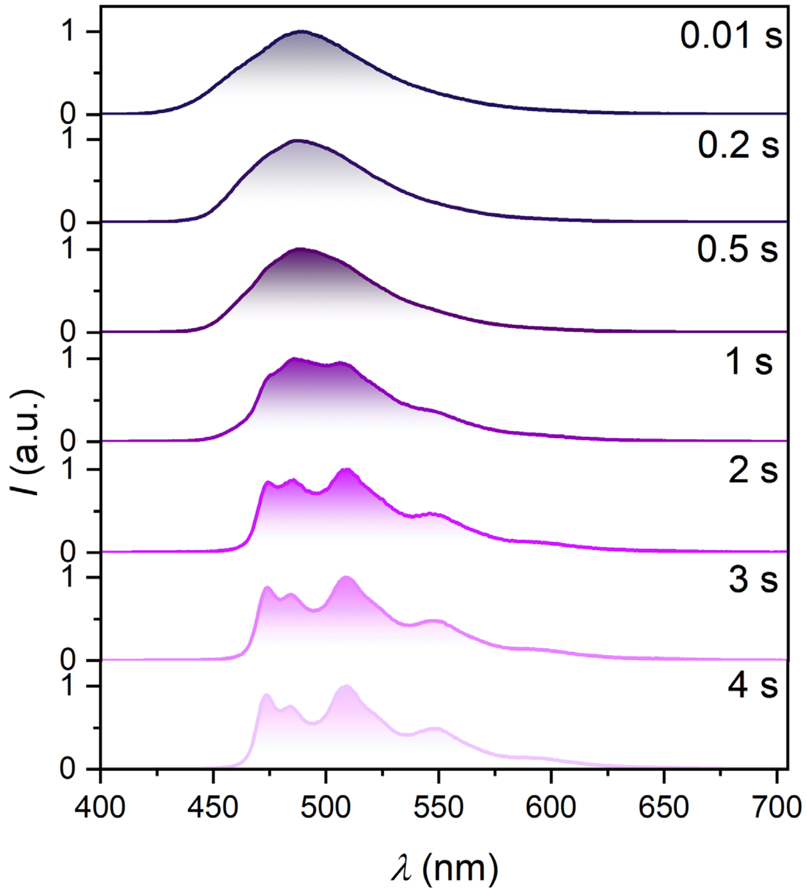

Figure S11: Time-gated 77 K emission spectra of **TX-Ntl-0** in frozen water (250 mM NaCl, 10 mM Na-Pi buffer) after 355 nm laser pulse excitation. Time delays to the excitation were set between 0.01 and 4 s. The integration window ranged from 100 ms to 300 ms.

The difference in triplet energy between $^3TX_{DNA}$ and $^3Ntl_{DNA}$ was estimated on the basis of room temperature (293 K) TA spectra and 77 K emission spectra of **TX-Ntl-0** according to the Boltzmann distribution expressed below:[18–20]

$$\ln\left(\frac{n_2}{n_1}\right) = -\frac{1}{T}\cdot\frac{\Delta E}{R} \qquad (2)$$

Where $\frac{n_2}{n_1}$ is the ratio between $^3TX_{DNA}$ and $^3Ntl_{DNA}$ in the equilibrated state, T is the temperature, R is the gas constant, and ΔE is the energy difference. The ratio $\frac{n_2}{n_1}$ was estimated at 293 K based on the reduction of the triplet absorption maximum of TX_{DNA} at 612 nm (Figure 2A, main paper) to be 0.61 (i.e., in the TTET equilibrium), assuming that $^3TX_{DNA}$ is not deactivated by alternative loss channels. The phosphorescence spectra of $^3TX_{DNA}$ and $^3Ntl_{DNA}$ at 77 K are almost entirely superimposed making it difficult to determine an exact ratio. However, assuming that the phosphorescence quantum yield is similar for both emitters and that only $^3TX_{DNA}$ emits at 450 nm, we estimated a ratio of $\frac{n_2}{n_1} = 0.05$ as an upper limit. Using these ratios, we obtained an energy difference of $\Delta E \sim 0.02$ eV. Given that the triplet state energy of $^3Ntl_{DNA}$ was determined at 2.61 eV from the fine-structured phosphorescence spectrum, we estimated the triplet state energy of $^3TX_{DNA}$ at ~ 2.63 eV.

5. Further discussion of the energy transfer mechanism

Bridge-mediated triplet-triplet energy transfer can occur through various coupling mechanisms possibly involving virtual intermediate states.[21–23] The assumption that the TTET attenuation factor is the sum of the individual electron and hole contributions no longer holds when electron and hole transfer do not occur concertedly forming intermediate charge transfer (CT) states or bridge-localized excited states.[22–25] Instead, the attenuation factor is more likely governed by the rate-limiting initial transfer step, i.e. either electron or hole transfer reaching a virtual CT state, followed by a rapid complementary electron/hole transfer.[23]

These virtual states are essentially undetectable using conventional spectroscopic techniques. Nonetheless, as with standard electron (or hole) transfer, by semi-classical theory the attenuation factor can be related to the effective barrier height, with the transfer rate being proportional to the electronic coupling matrix element.[21] This prediction was shown to be in good agreement with experimental values. [26]

$$\Delta E_{\mathrm{eff}} = \left(\frac{\hbar}{8m_e}\right)\beta^2 = \left(0.952\ \mathrm{eV}\ \text{Å}^2\right)\beta^2 \qquad (3)$$

If virtual CT states dominate the Dexter pathway, the theoretically determined barrier height would be expected to approximate the experimental value. With the attenuation factor determined in our study (1.15 Å^{-1}), we calculate an effective barrier height of 1.26 eV using equation (3). Given that this barrier height is defined by the energy gap between the donor (~2.63 eV for TX_{DNA}) and the bridge (thymine), this value would exceed the triplet energy of even free thymine.[27] In fact, literature reports place the triplet energy of thymine in poly A-T sequences at 3.0 eV, which would suggest a much weaker distance dependence than observed.[28] This discrepancy indicates that bridge-mediated coupling and virtual CT states play a negligible role in the TTET pathway in our study, and it rather suggests a concerted transfer mechanism.[22,23,29]

6. Images of ^{1}H, ^{13}C and ^{31}P NMR spectra and of MS analyses

Compound 2a

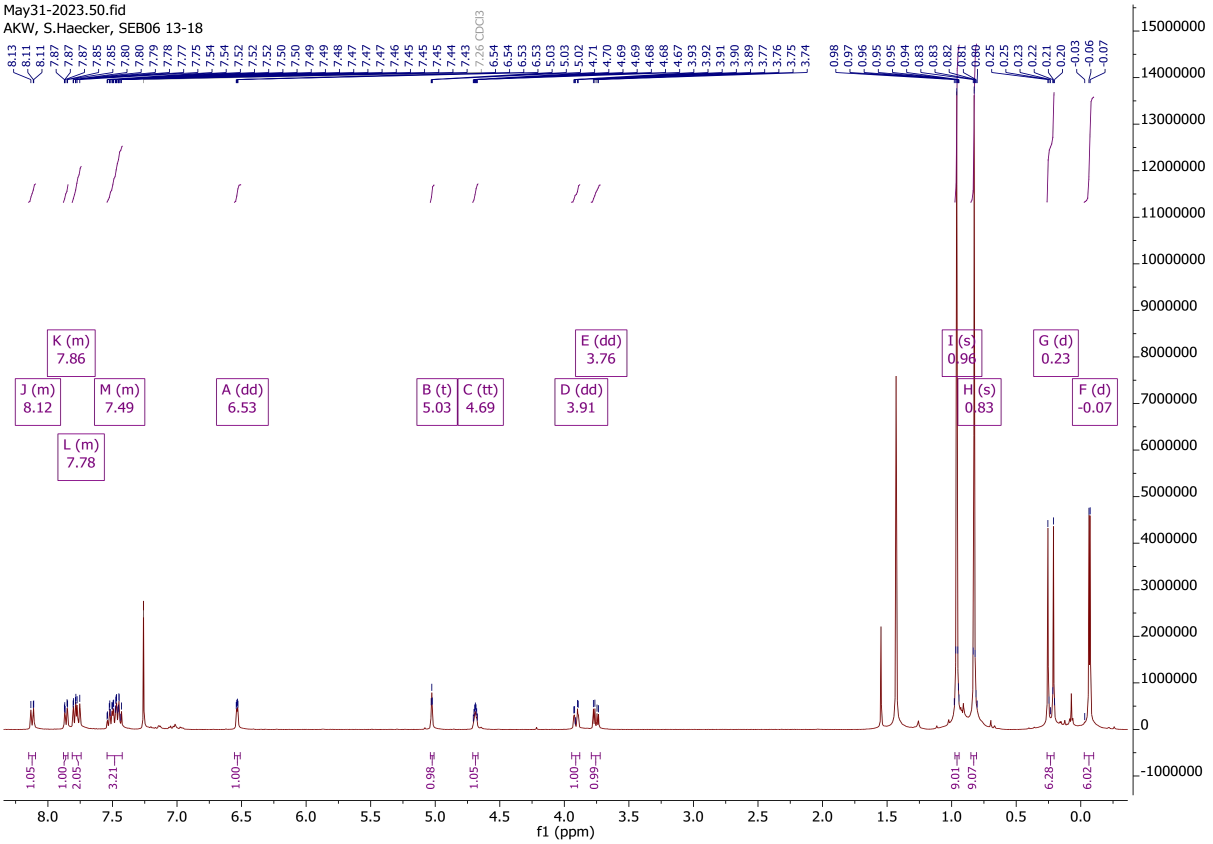

Figure S12: ^{1}H NMR (400 MHz, $CDCl_3$) of **2a**.

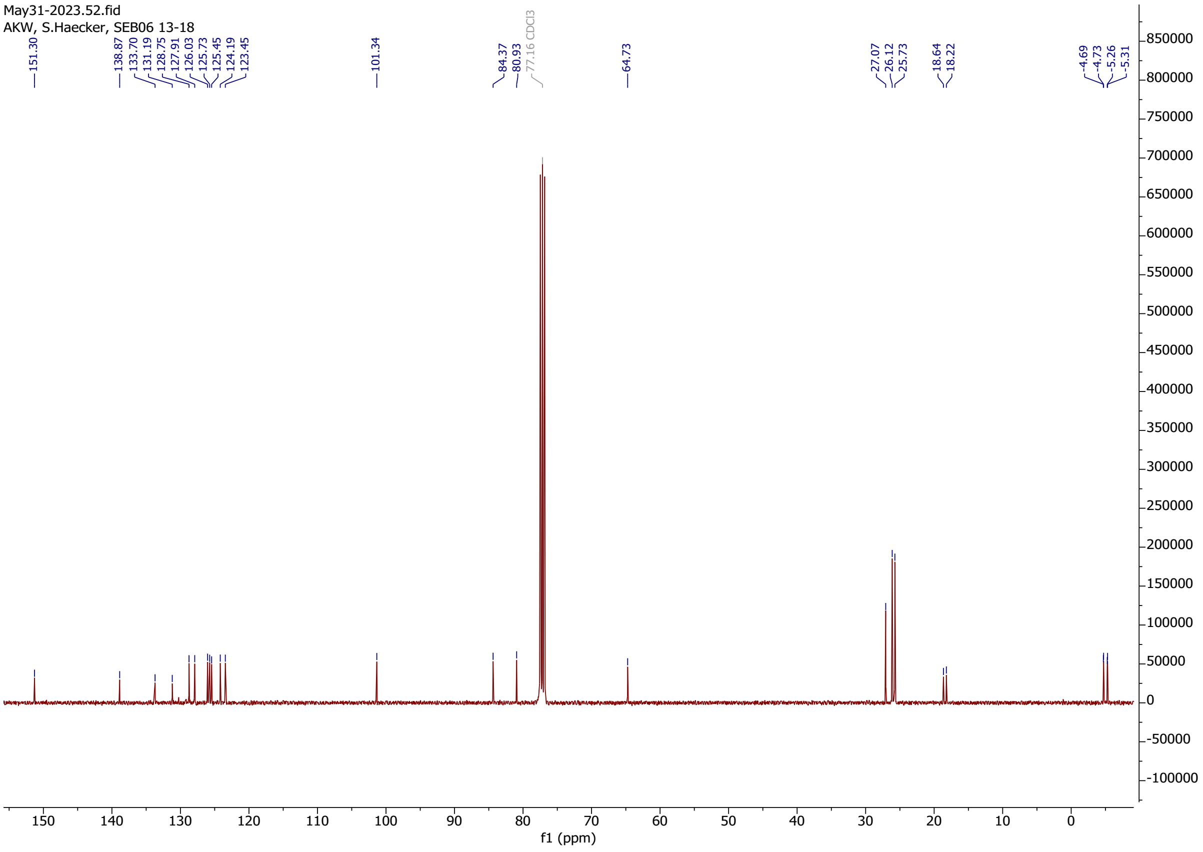

Figure S13: ^{13}C NMR (101 MHz, $CDCl_3$) of **2a**.

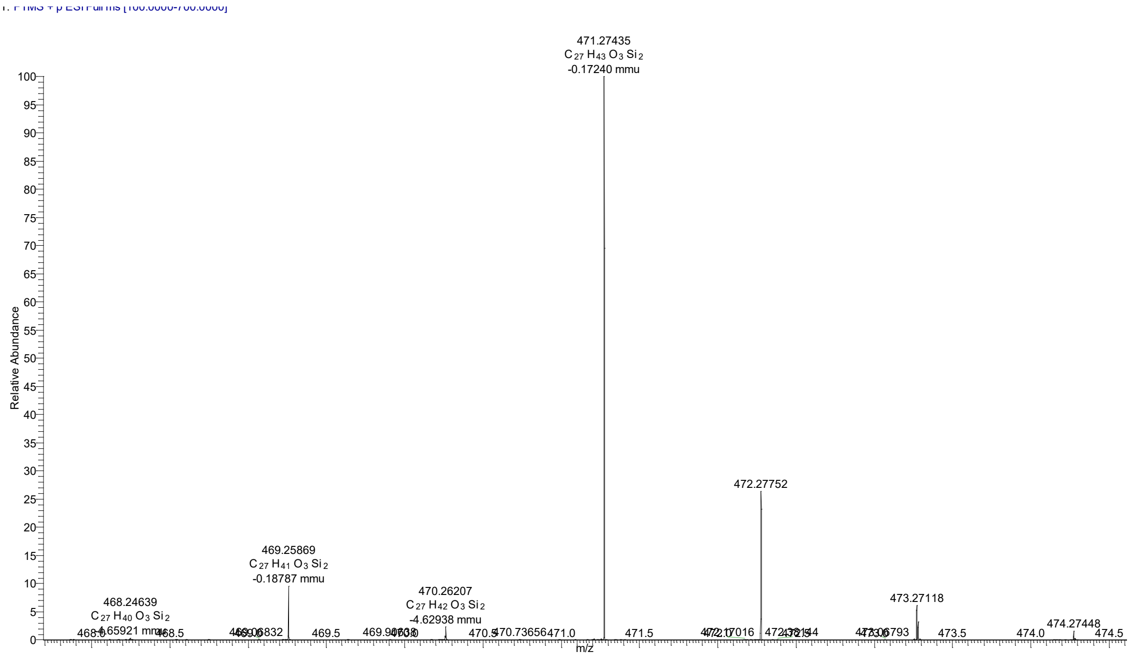

Figure S14: HR ESI-MS analysis of **2a**.

Compound 2b

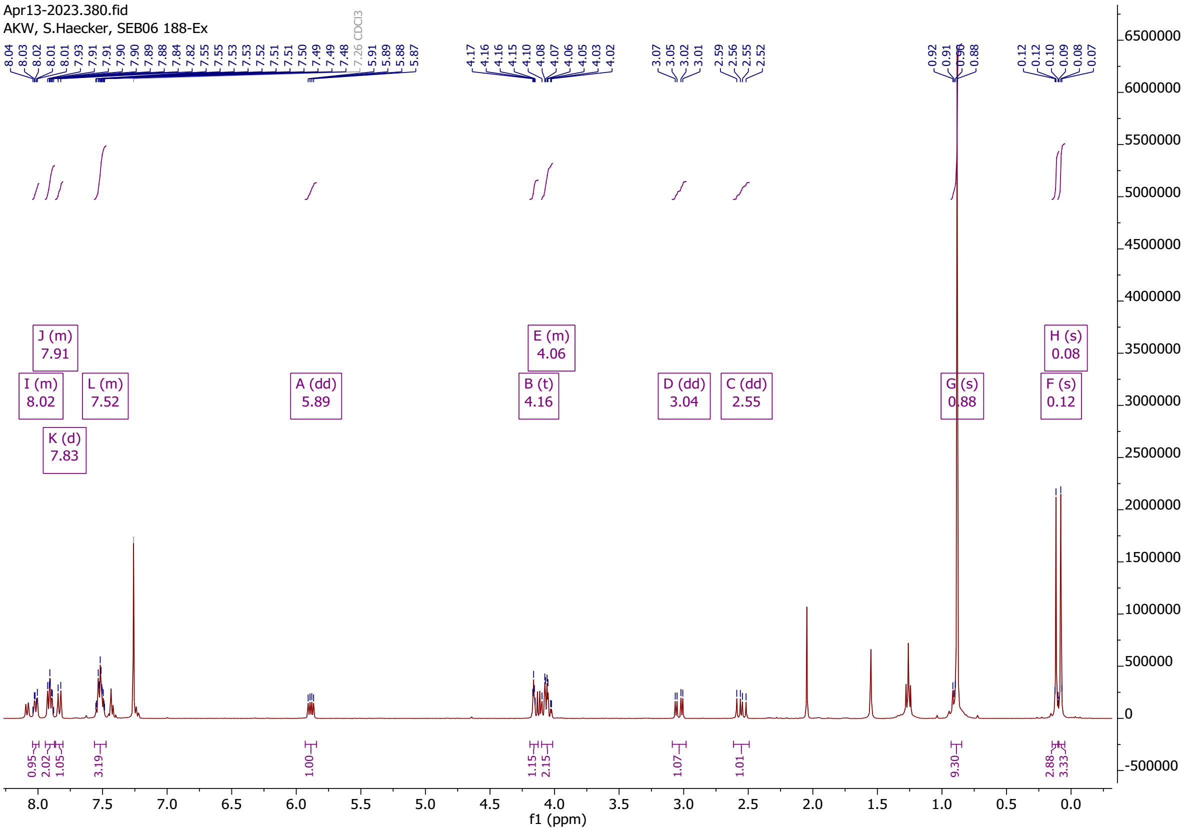

Figure S15: ^{1}H NMR (400 MHz, $CDCl_3$) of **2b**.

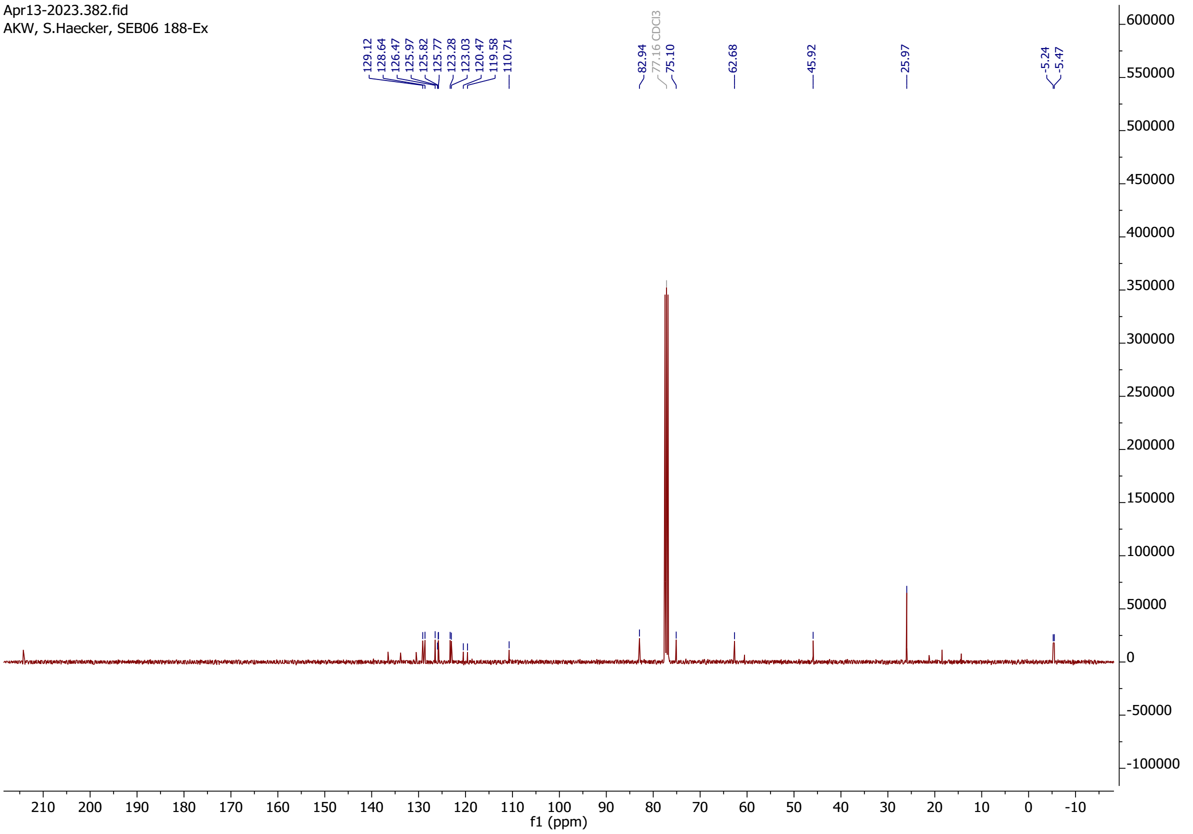

Figure S16: ^{13}C NMR (101 MHz, $CDCl_3$) of **2b**.

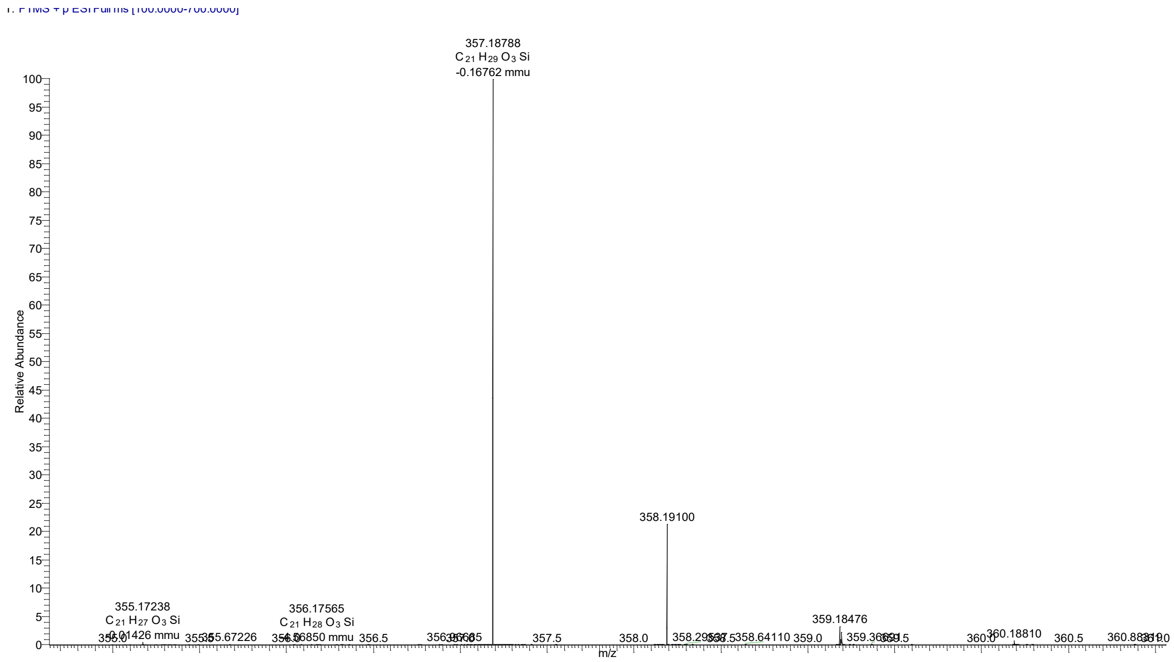

Figure S17: HR ESI-MS analysis of **2b**.

Compound 3

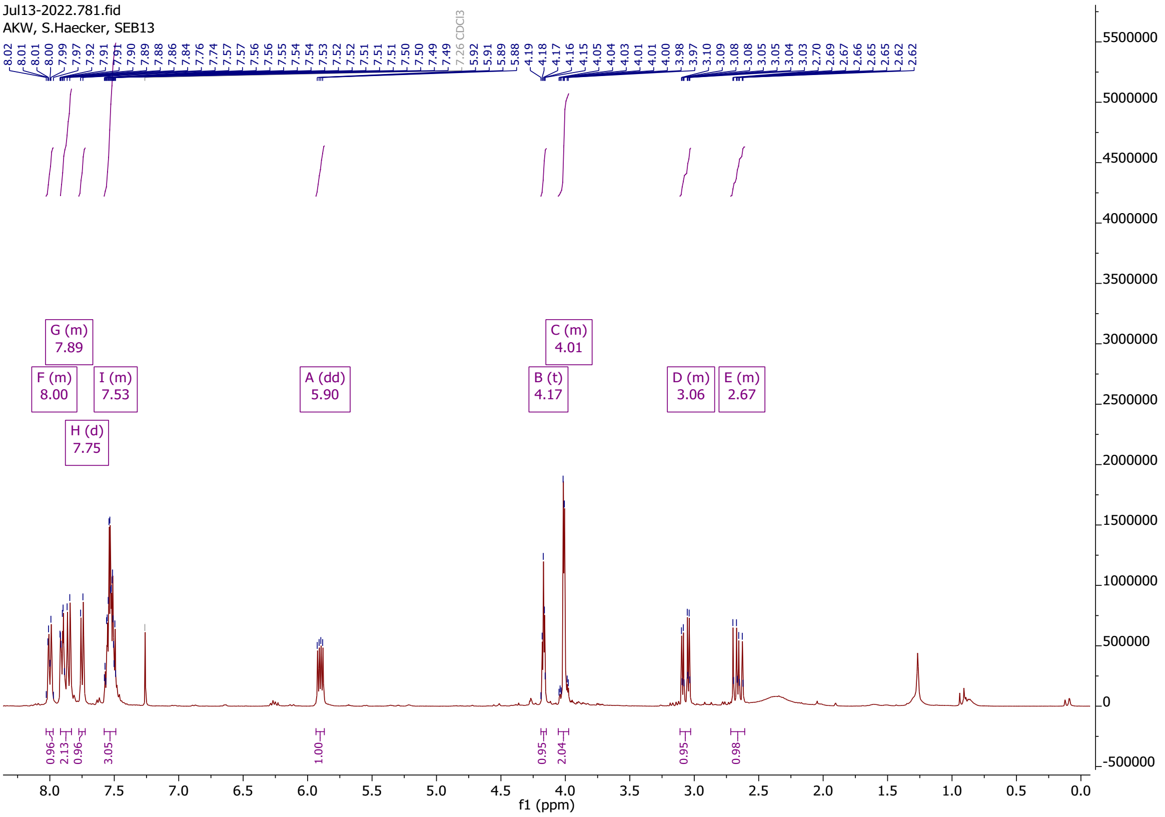

Figure S18: ^{1}H NMR (400 MHz, $CDCl_3$) of **3**.

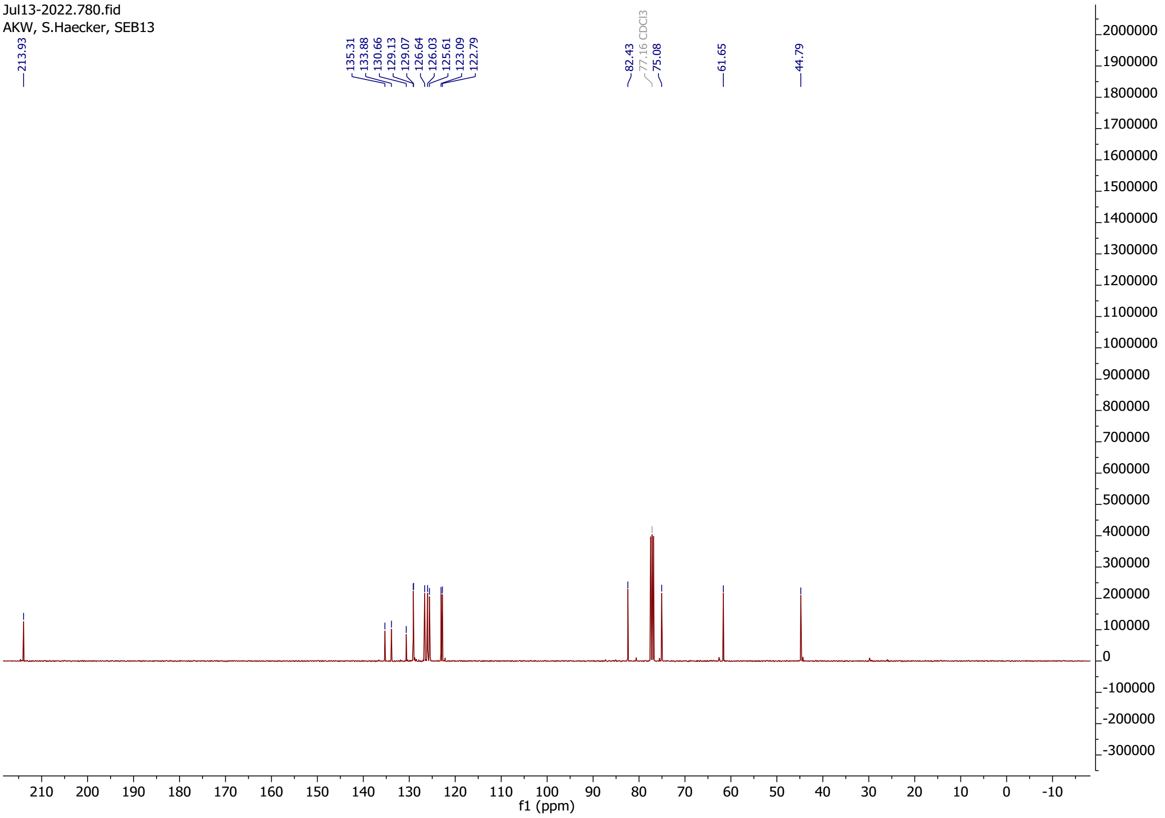

Figure S19: ^{13}C NMR (101 MHz, $CDCl_3$) of **3**.

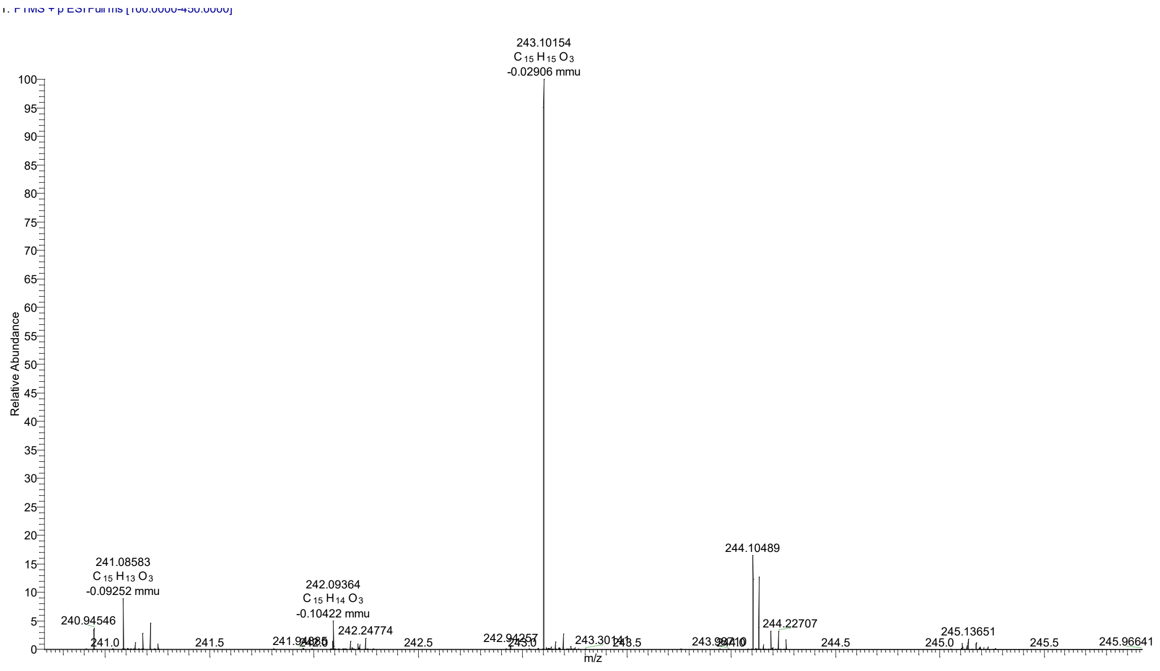

Figure S20: HR ESI-MS analysis of **3**.

Compound 4

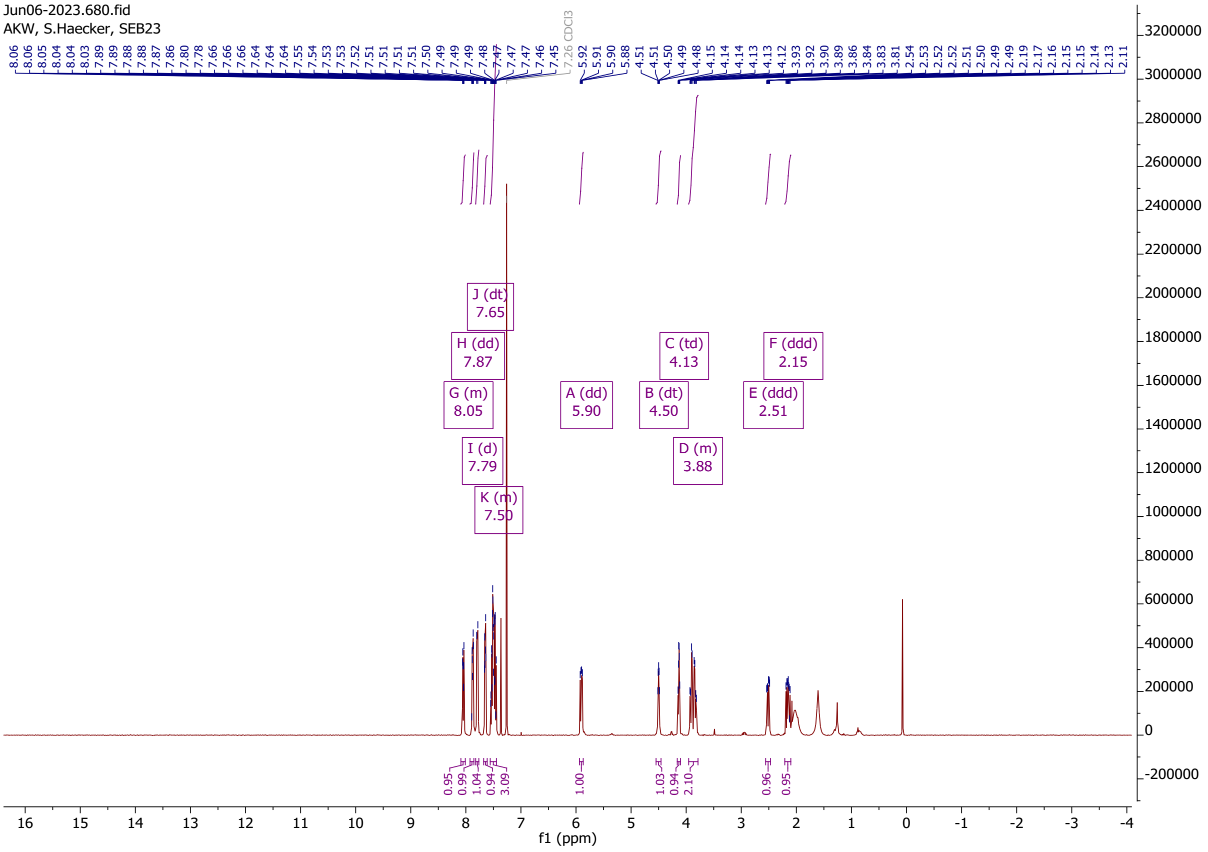

Figure S21: ^{1}H NMR (400 MHz, $CDCl_3$) of **4**.

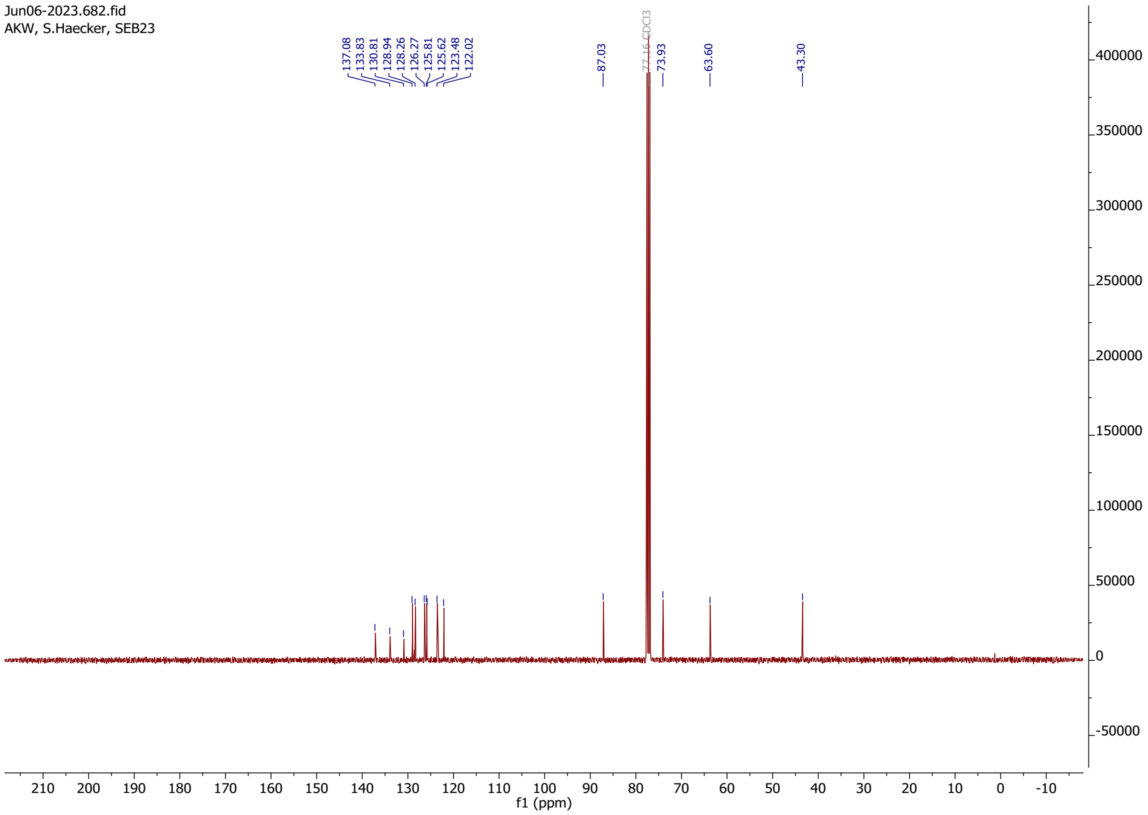

Figure S22: ^{13}C NMR (101 MHz, $CDCl_3$) of **4**.

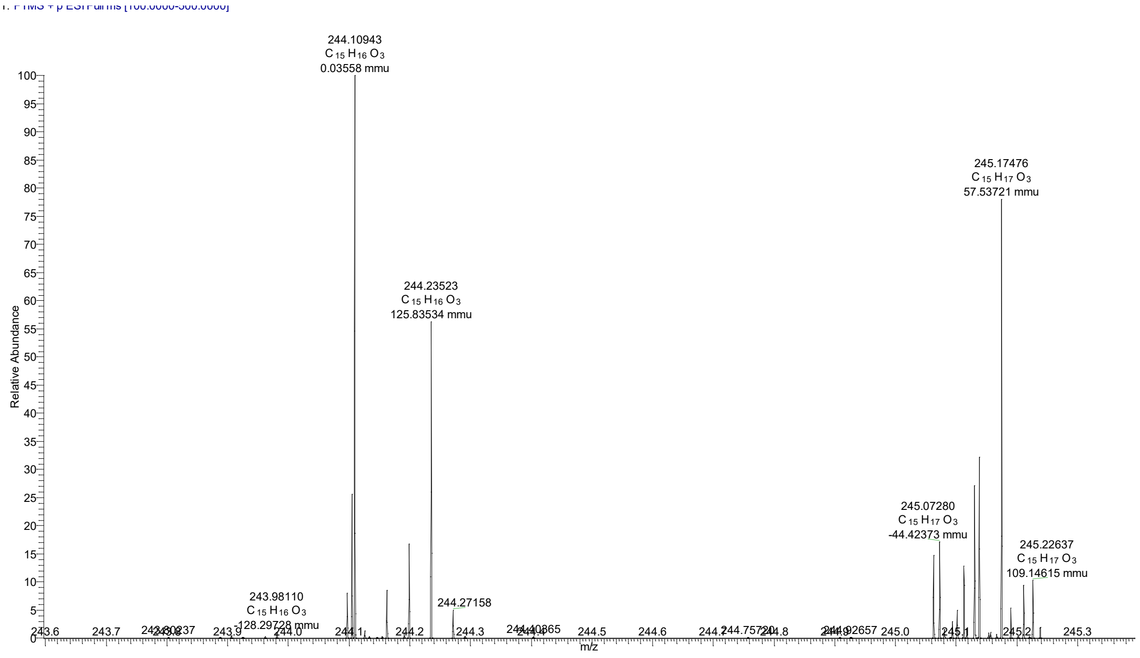

Figure S23: HR ESI-MS analysis of **4**.

Compound 5

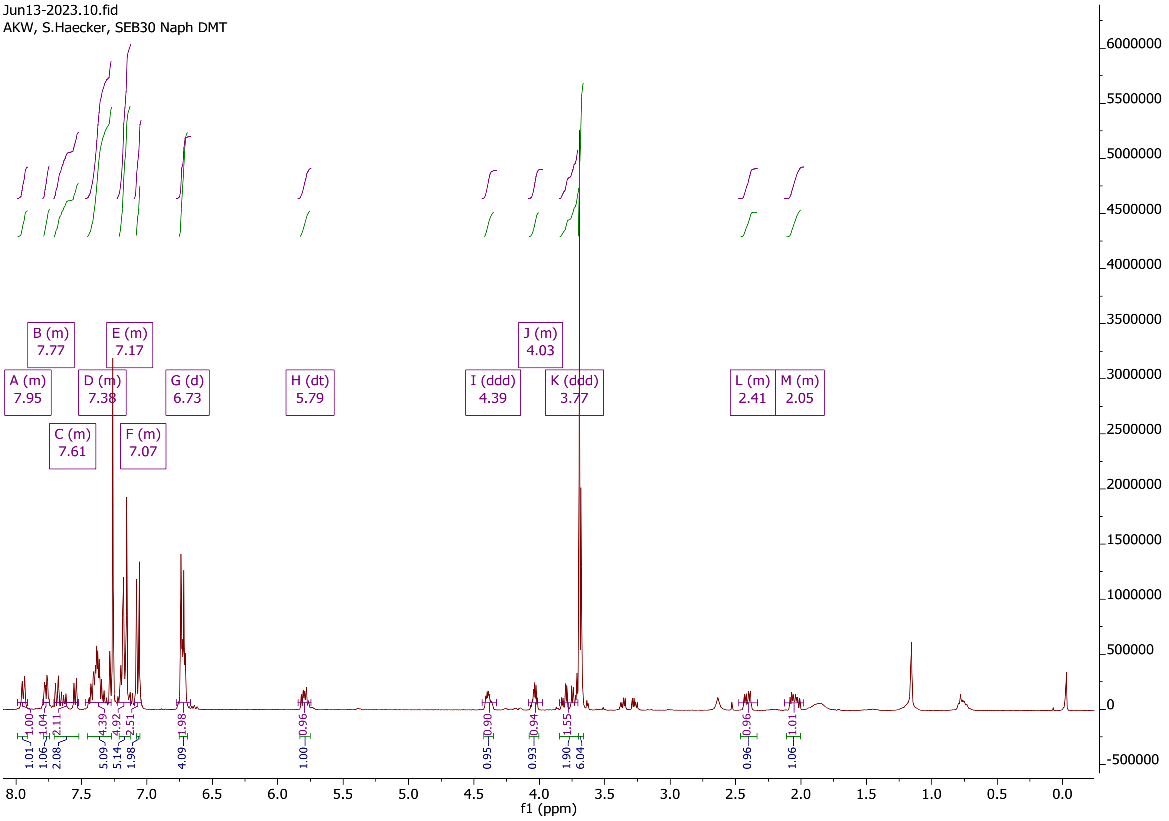

Figure S24: ^{1}H NMR (400 MHz, $CDCl_3$) of **5**.

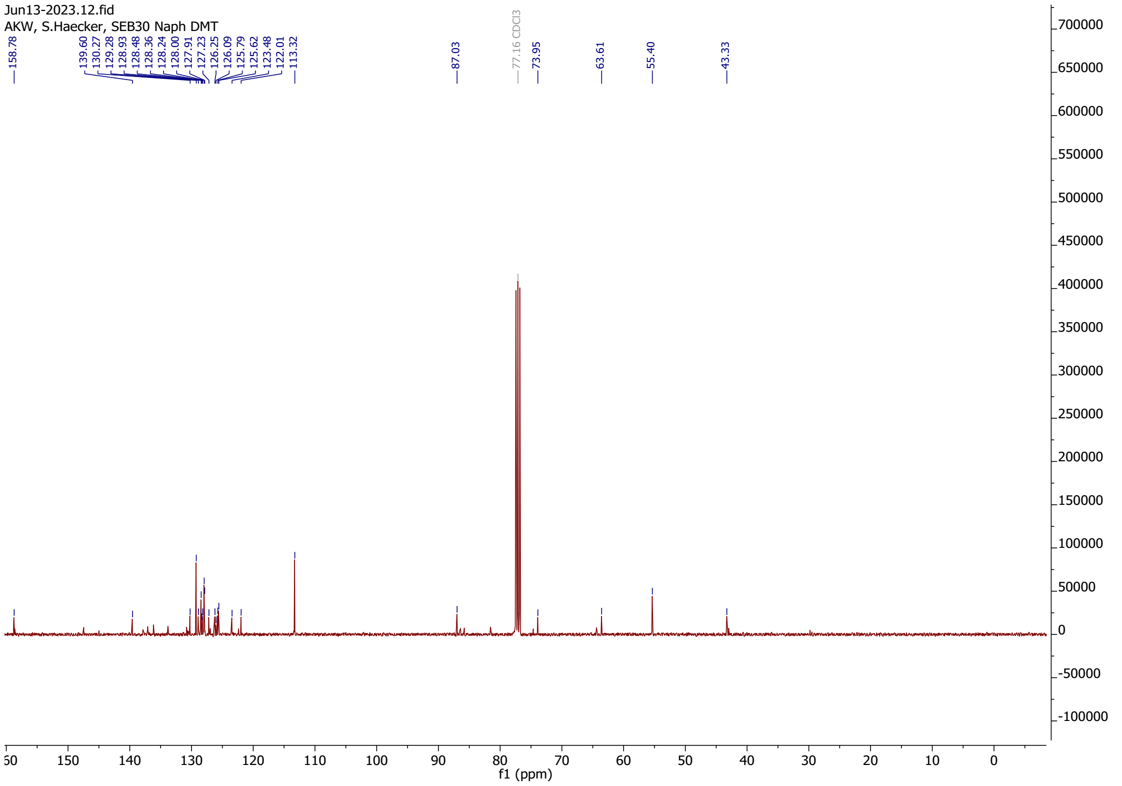

Figure S25: ^{13}C NMR (101 MHz, $CDCl_3$) of **5**.

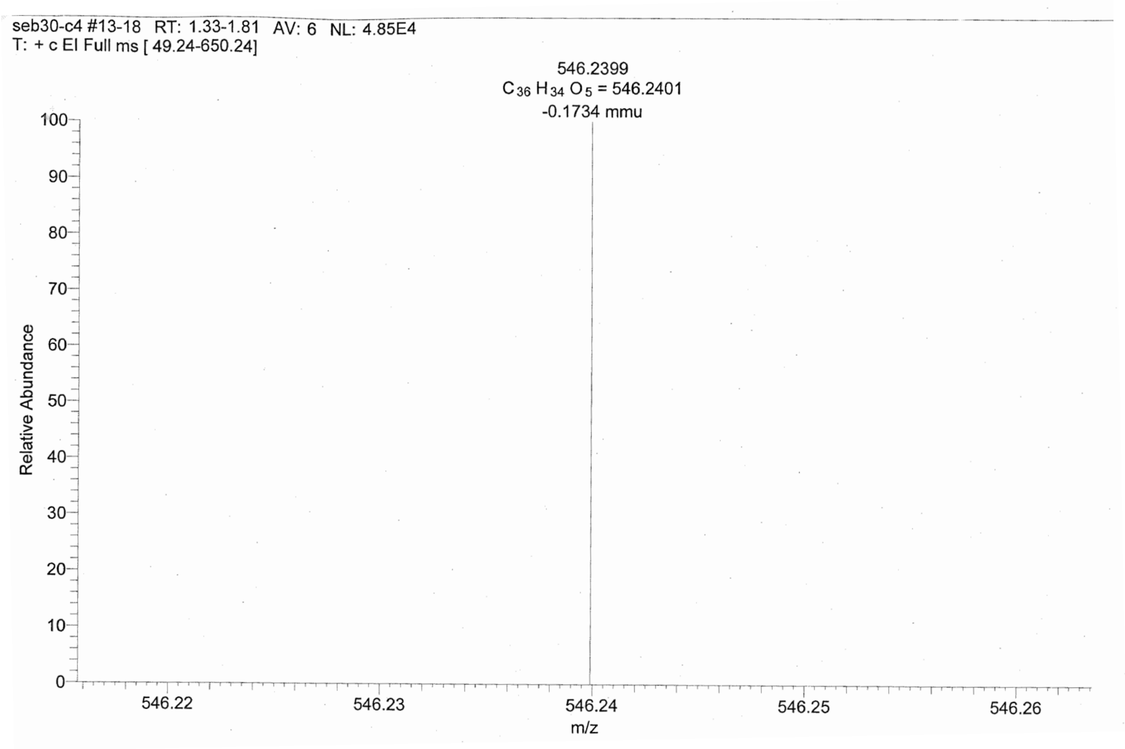

Figure S26: HR-EI-MS analysis of **5**.

Compound 6

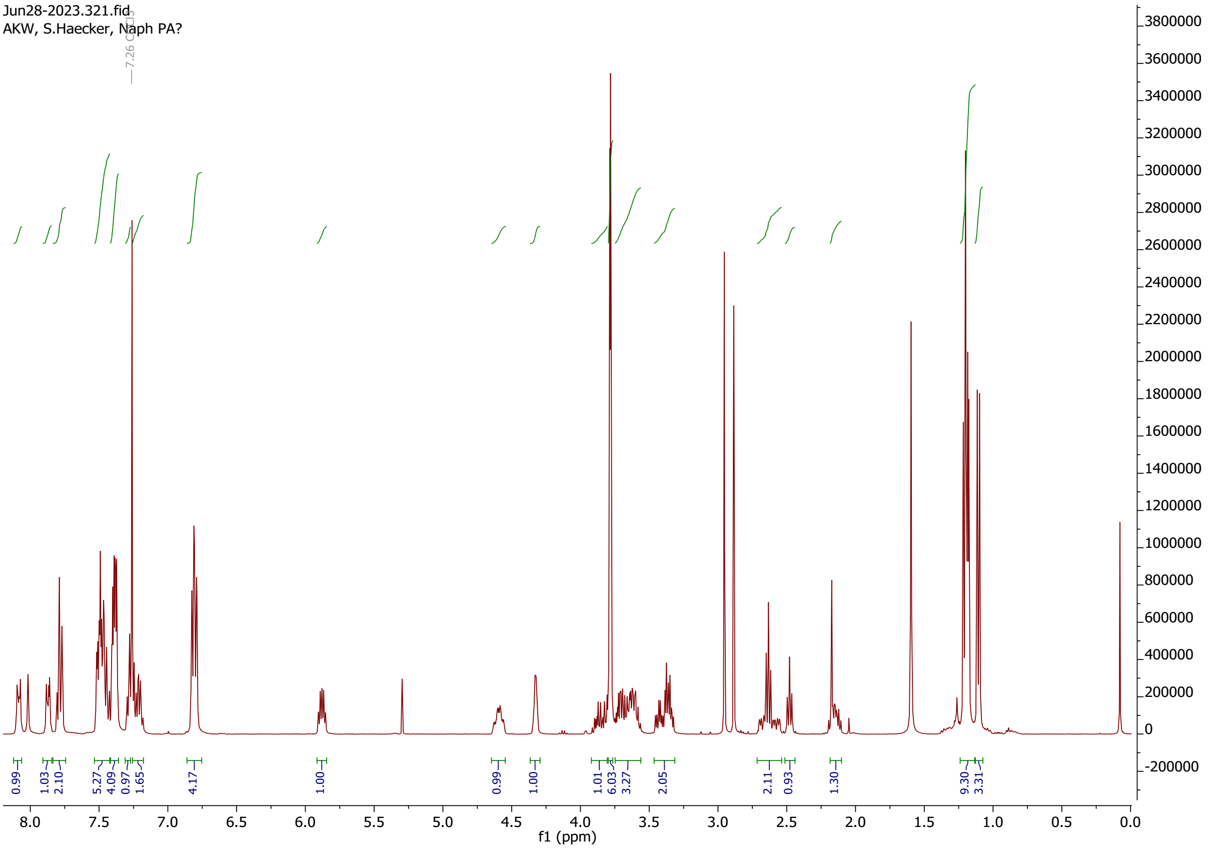

Figure S27: ^{1}H NMR (400 MHz, $CDCl_3$) of **6**.

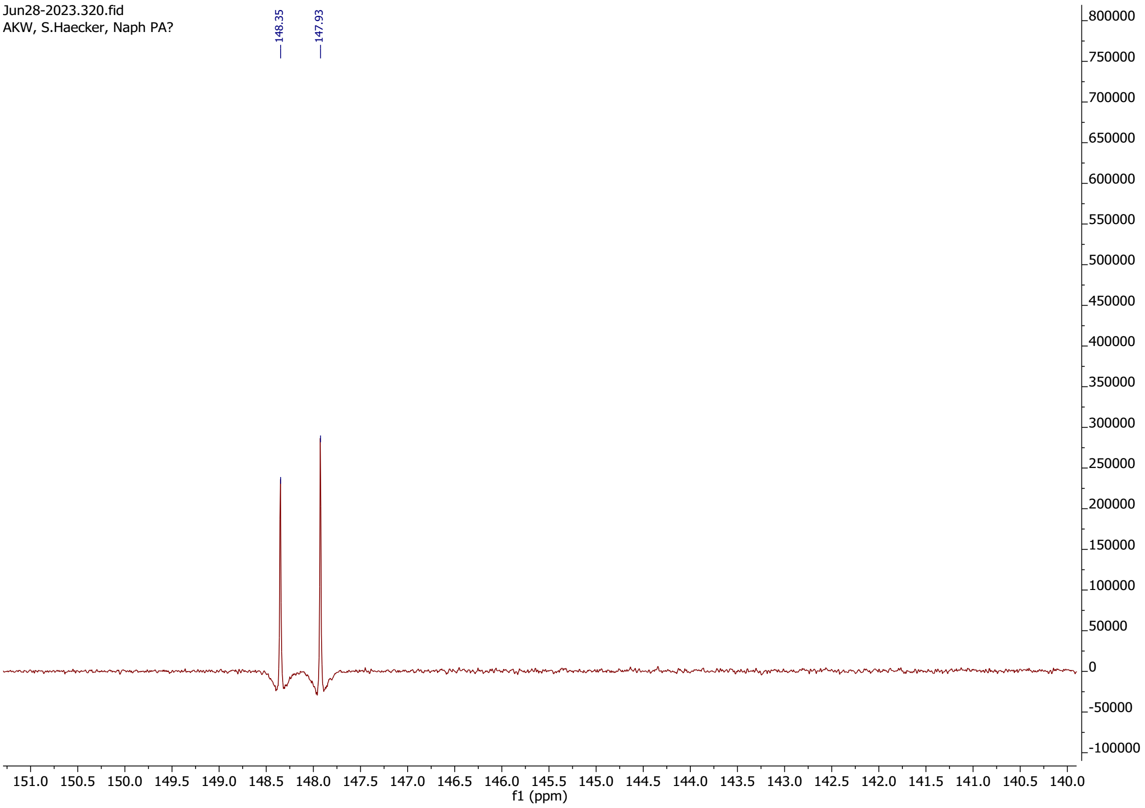

Figure S28: ^{31}P NMR (162 MHz, $CDCl_3$) of **6**.

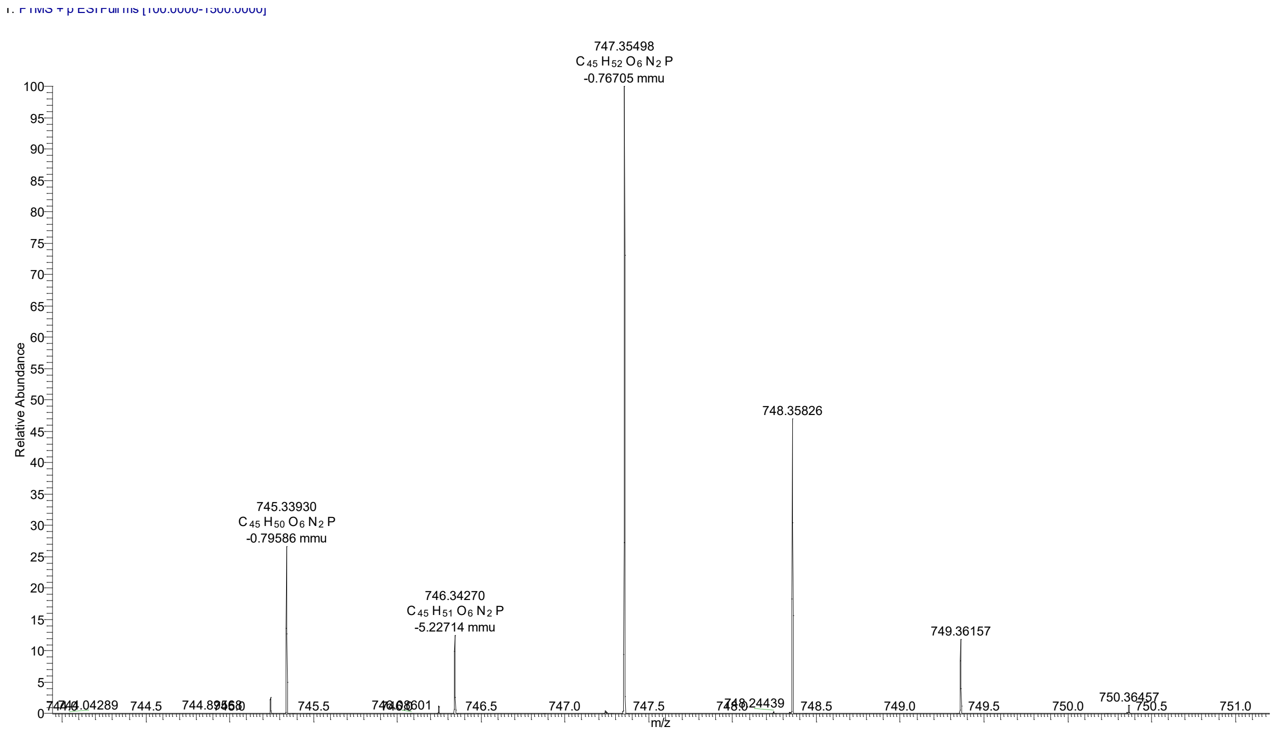

Figure S29: HR ESI-MS analysis of **6**.

7. DNA analyses and melting temperatures

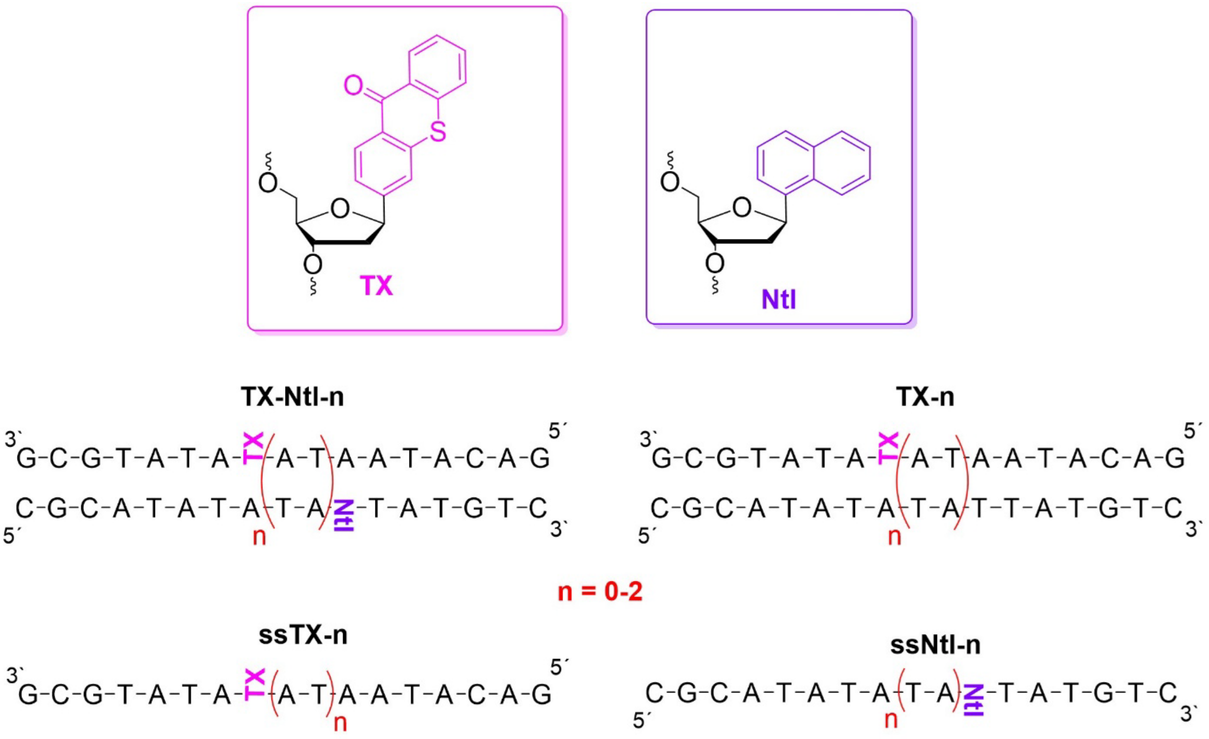

Figure S30: Overview of Thioxanthone (**TX**)- and Naphthalene (**Ntl**)-modified single-stranded oligonucleotides (**ssTX-n** and **ssNtl-n**), as well as the hybridized DNA double-strands (**TX-Ntl-n** and **TX-n**).

The extinction coefficients ε_{260} of the single strand oligonucleotides (**ssTX-n** and **ssNtl-n**) were determined using the following equation in $M^{-1}\cdot cm^{-1}$:

$$\varepsilon_{260} = \varepsilon_{260}X + (nA^* \varepsilon_A(15400) + nT^* \varepsilon_T(8800) + nG^* \varepsilon_G(11700) + nC^* \varepsilon_C(7300))^* 0.9 \quad (4)$$

Table S4: Extinction coefficient and MS (MALDI-TOF) data of the synthesized TX- and Ntl- modified single strands **ssTX/Ntl-n**.

**ssTX/Ntl-n**	ε_{260} nm [$M^{-1}\cdot cm^{-1}$]	Mass calcd. [Da]	Mass found [Da]
**ssTX-0**	189850	4684	4683
**ssTX-1**	211630	5301	5298
**ssTX-2**	233410	5918	5920
**ssNtl-0**	139610	4542	4542
**ssNtl-1**	161390	5159	5161
**ssNtl-2**	183170	5776	5776

Table S5 Melting temperatures of the annealed DNA: **TX-n** and **TX-Ntl-n**.

**DNA**	Melting temperature [°C]
**TX-0**	52°C
**TX-Ntl-0**	48°C
**TX-1**	54°C
**TX-Ntl-1**	46°C
**TX-2**	56°c
**TX-Ntl-2**	50°C

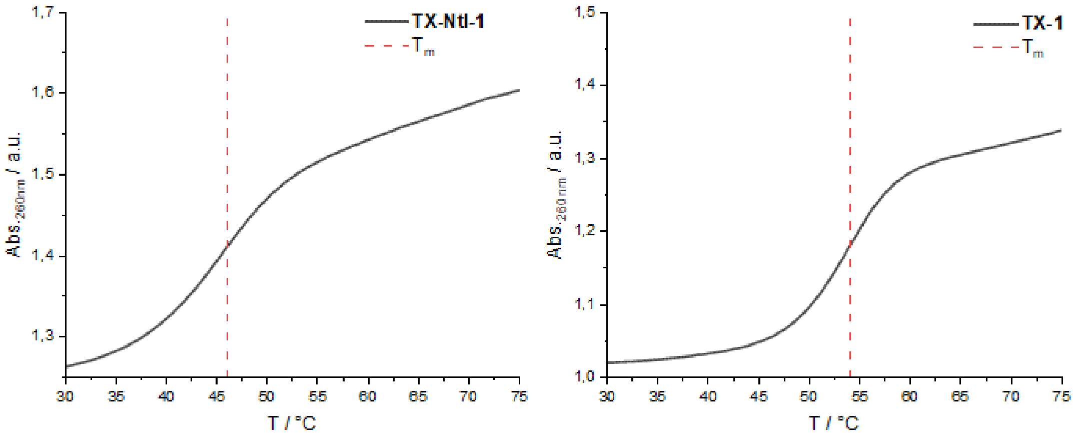

Figure S31: Exemplary melting curve at 260 nm for **TX-1** (right) and **TX-Ntl-1** (left).

8. Images of MS and HPLC analyses of the DNA

The **ssTX-n** oligonucleotides have been previously described and published in the literature.[30]

ssNtl-0:

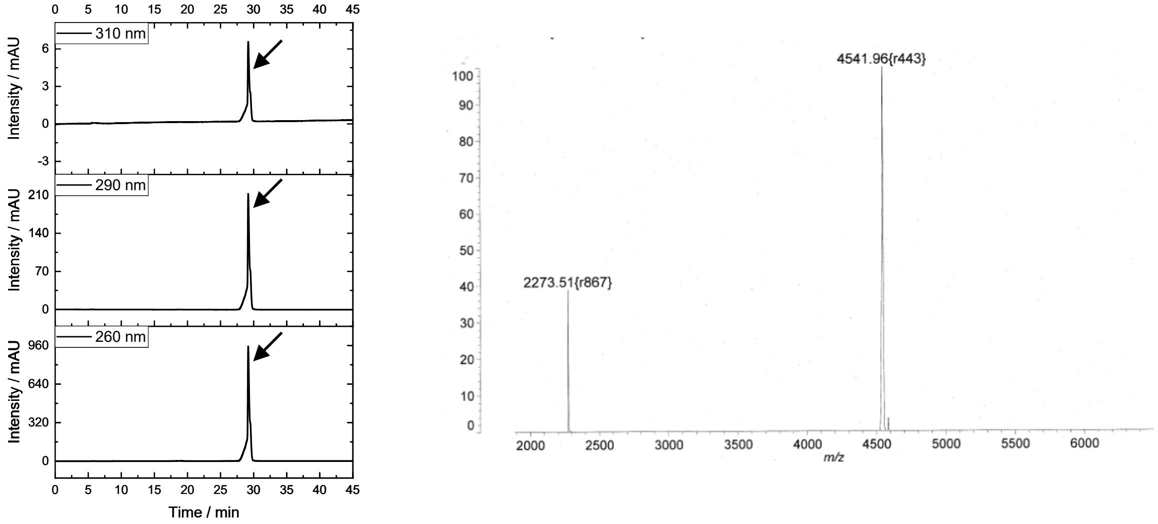

Figure S32: Left: HPLC analysis (260 nm, 290 nm and 310 nm) of **ssNtl-0**. Right: MALDI-TOF-MS analysis of **ssNtl-0**.

ssNtl-1:

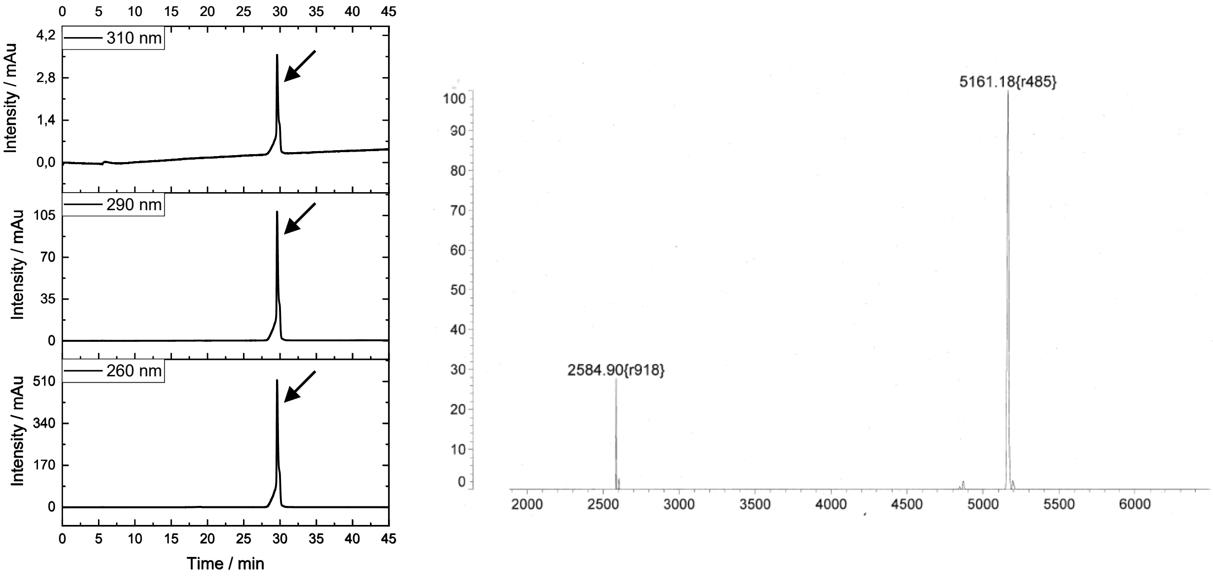

Figure S33: Left: HPLC analysis (260 nm, 290 nm and 310 nm) of **ssNtl-1**. Right: MALDI-TOF-MS analysis of **ssNtl-1**.

ssNtl-2:

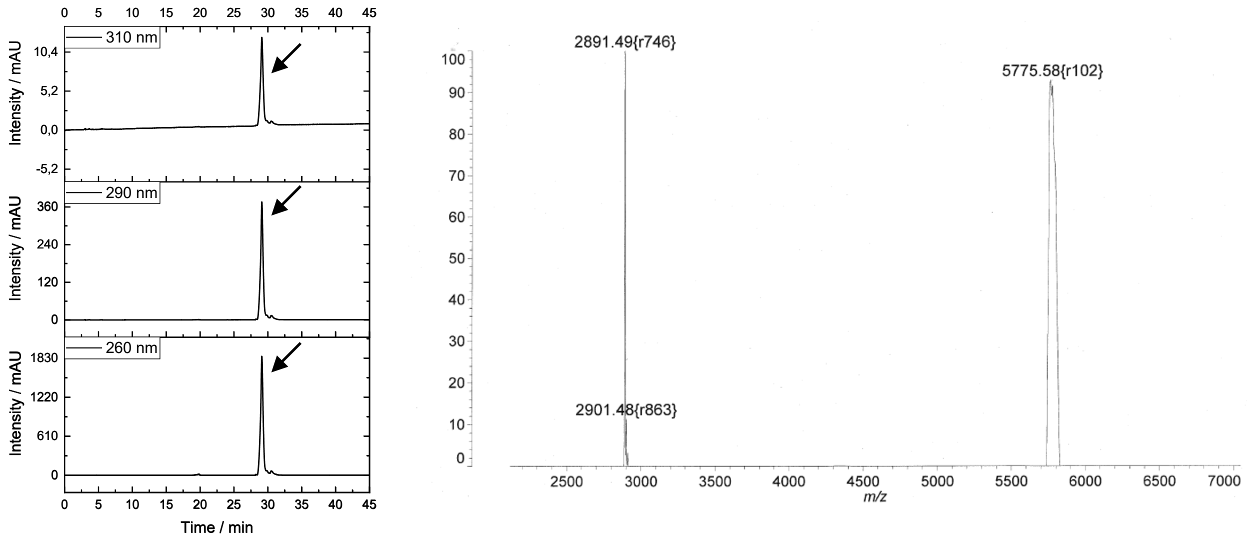

Figure S34: Left: HPLC analysis (260 nm, 290 nm and 310 nm) of **ssNtl-2**. Right: MALDI-TOF-MS analysis of **ssNtl-2**.

9. References

(1) DeBoer, C. D.; Schlessinger, R. H. Rate of the Self-Quenching Reaction for Thioxanthone Triplets. *J. Am. Chem. Soc.* **1972**, *94* (2), 655–656. https://doi.org/10.1021/ja00757a067.

(2) Gudem, M.; Kowalewski, M. Triplet-triplet Annihilation Dynamics of Naphthalene. *Chem. – Eur. J.* **2022**, *28* (40), e202200781. https://doi.org/10.1002/chem.202200781.

(3) Tfibel, F.; Lindqvist, L. Excited Singlet Yield in T-T Annihilation, a Comparative Study of Naphthalene and Anthracene in Solution. *Chem. Phys.* **1975**, *10* (2–3), 471–478. https://doi.org/10.1016/0301-0104(75)87059-5.

(4) Zähringer, T. J. B.; Wienhold, M.; Gilmour, R.; Kerzig, C. Direct Observation of Triplet States in the Isomerization of Alkenylboronates by Energy Transfer Catalysis. *J. Am. Chem. Soc.* **2023**, *145* (39), 21576–21586. https://doi.org/10.1021/jacs.3c07678.

(5) Montalti, M.; Credi, A.; Prodi, L.; Gandolfi, M. T. *Handbook of Photochemistry*, 0 ed.; CRC Press, 2006. https://doi.org/10.1201/9781420015195.

(6) Bieri, O.; Wirz, J.; Hellrung, B.; Schutkowski, M.; Drewello, M.; Kiefhaber, T. The Speed Limit for Protein Folding Measured by Triplet–Triplet Energy Transfer. *Proc. Natl. Acad. Sci.* **1999**, *96* (17), 9597–9601. https://doi.org/10.1073/pnas.96.17.9597.

(7) Klán, P.; Wirz, J. *Photochemistry of Organic Compounds: From Concepts to Practice*; Wiley: Chichester, West Sussex, U.K, 2009.

(8) Kerzig, C.; Goez, M. Generating Hydrated Electrons through Photoredox Catalysis with 9-Anthrolate. *Phys. Chem. Chem. Phys.* **2015**, *17* (21), 13829–13836. https://doi.org/10.1039/C5CP01711D.

(9) Wallace, S. C.; Grätzel, M.; Thomas, J. K. Laser Photoionization of Aromatic Hydrocarbons in Micellar Solution. *Chem. Phys. Lett.* **1973**, *23* (3), 359–362. https://doi.org/10.1016/0009-2614(73)85097-3.

(10) Dhenadhayalan, N.; Selvaraju, C. Role of Photoionization on the Dynamics and Mechanism of Photoinduced Electron Transfer Reaction of Coumarin 307 in Micelles. *J. Phys. Chem. B* **2012**, *116* (16), 4908–4920. https://doi.org/10.1021/jp301412c.

(11) Guéron, M.; Eisinger, J.; Shulman, R. G. Excited States of Nucleotides and Singlet Energy Transfer in Polynucleotides. *J. Chem. Phys.* **1967**, *47* (10), 4077–4091. https://doi.org/10.1063/1.1701580.

(12) Allen, N. S. *Photopolymerisation and Photoimaging Science and Technology*; Springer Netherlands: Dordrecht, 1989. https://doi.org/10.1007/978-94-009-1127-7.

(13) Li, H.; Yao, S.; Zuo, Z.; Wang, W.; Zhang, J.; Lin, N. Characterization of the Reactive Intermediates in Laser Flash Photolysis of Adenine, Adenosine and dAMP Using Acetone as Photosensitizer. *J. Photochem. Photobiol. B* **1995**, *28* (1), 65–70. https://doi.org/10.1016/1011-1344(94)07095-6.

(14) Krystkowiak, E.; Maciejewski, A.; Kubicki, J. Spectral and Photophysical Properties of Thioxanthone in Protic and Aprotic Solvents: The Role of Hydrogen Bonds in S_1 -Thioxanthone Deactivation. *ChemPhysChem* **2006**, *7* (3), 597–606. https://doi.org/10.1002/cphc.200500375.

(15) Dossot, M.; Allonas, X.; Jacques, P. Singlet Exciplexes between a Thioxanthone Derivative and Substituted Aromatic Quenchers: Role of the Resonance Integral. *Chem. – Eur. J.* **2005**, *11* (6), 1763–1770. https://doi.org/10.1002/chem.200400269.

(16) Burget, D.; Jacques, P. Dramatic Solvent Effects on Thioxanthone Fluorescence Lifetime. *J. Lumin.* **1992**, *54* (3), 177–181. https://doi.org/10.1016/0022-2313(92)90006-U.

(17) MacArthur, C. G. Solubility of Oxygen in Salt Solutions and the Hydrates of These Salts. *J. Phys. Chem.* **1916**, *20* (6), 495–502. https://doi.org/10.1021/j150168a003.

(18) Geitenbeek, R. G.; Prins, P. T.; Albrecht, W.; Van Blaaderen, A.; Weckhuysen, B. M.; Meijerink, A. $NaYF_4$:Er^{3+} ,Yb^{3+} /SiO_2 Core/Shell Upconverting Nanocrystals for Luminescence Thermometry up to 900 K. *J. Phys. Chem. C* **2017**, *121* (6), 3503–3510. https://doi.org/10.1021/acs.jpcc.6b10279.

(19) Back, M.; Ueda, J.; Brik, M. G.; Tanabe, S. Pushing the Limit of Boltzmann Distribution in Cr^{3+} -Doped $CaHfO_3$ for Cryogenic Thermometry. *ACS Appl. Mater. Interfaces* **2020**, *12* (34), 38325–38332. https://doi.org/10.1021/acsami.0c08965.

(20) Yarnell, J. E.; Wells, K. A.; Palmer, J. R.; Breaux, J. M.; Castellano, F. N. Excited-State Triplet Equilibria in a Series of Re(I)-Naphthalimide Bichromophores. *J. Phys. Chem. B* **2019**, *123* (35), 7611–7627. https://doi.org/10.1021/acs.jpcb.9b05688.
(21) Gray, H. B.; Winkler, J. R. Long-Range Electron Transfer. *Proc. Natl. Acad. Sci.* **2005**, *102* (10), 3534–3539. https://doi.org/10.1073/pnas.0408029102.
(22) Skourtis, S. S.; Liu, C.; Antoniou, P.; Virshup, A. M.; Beratan, D. N. Dexter Energy Transfer Pathways. *Proc. Natl. Acad. Sci.* **2016**, *113* (29), 8115–8120. https://doi.org/10.1073/pnas.1517189113.
(23) Lai, R.; Liu, Y.; Luo, X.; Chen, L.; Han, Y.; Lv, M.; Liang, G.; Chen, J.; Zhang, C.; Di, D.; Scholes, G. D.; Castellano, F. N.; Wu, K. Shallow Distance-Dependent Triplet Energy Migration Mediated by Endothermic Charge-Transfer. *Nat. Commun.* **2021**, *12* (1), 1532. https://doi.org/10.1038/s41467-021-21561-1.
(24) Closs, G. L.; Johnson, M. D.; Miller, J. R.; Piotrowiak, P. A Connection between Intramolecular Long-Range Electron, Hole, and Triplet Energy Transfers. *J. Am. Chem. Soc.* **1989**, *111* (10), 3751–3753. https://doi.org/10.1021/ja00192a044.
(25) Curutchet, C.; Voityuk, A. A. Distance Dependence of Triplet Energy Transfer in Water and Organic Solvents: A QM/MD Study. *J. Phys. Chem. C* **2012**, *116* (42), 22179–22185. https://doi.org/10.1021/jp306280y.
(26) Wenger, O. S. Photoinduced Electron and Energy Transfer in Phenylene Oligomers. *Chem. Soc. Rev.* **2011**, *40* (7), 3538. https://doi.org/10.1039/c1cs15044h.
(27) Wood, P. D.; Redmond, R. W. Triplet State Interactions between Nucleic Acid Bases in Solution at Room Temperature: Intermolecular Energy and Electron Transfer. *J. Am. Chem. Soc.* **1996**, *118* (18), 4256–4263. https://doi.org/10.1021/ja954340+.
(28) García-Messeguer, R.; Navarrete-Miguel, M.; Martí, S.; Tuñón, I.; Roca-Sanjuán, D. DNA Triplet Energies by Free Energy Perturbation Theory. *J. Chem. Theory Comput.* **2025**, *21* (3), 1353–1359. https://doi.org/10.1021/acs.jctc.4c01583.
(29) Luo, X.; Han, Y.; Chen, Z.; Li, Y.; Liang, G.; Liu, X.; Ding, T.; Nie, C.; Wang, M.; Castellano, F. N.; Wu, K. Mechanisms of Triplet Energy Transfer across the Inorganic Nanocrystal/Organic Molecule Interface. *Nat. Commun.* **2020**, *11* (1), 28. https://doi.org/10.1038/s41467-019-13951-3.
(30) Häcker, S.; Schrödter, M.; Kuhlmann, A.; Wagenknecht, H.-A. Probing of DNA Photochemistry with C-Nucleosides of Xanthones and Triphenylene as Photosensitizers To Study the Formation of Cyclobutane Pyrimidine Dimers. *JACS Au* **2023**, *3* (7), 1843–1850. https://doi.org/10.1021/jacsau.3c00167.
